# A comparison of shared patterns of differential gene expression and gene ontologies in response to water-stress in roots and leaves of four diverse genotypes of *Lolium* and *Festuca* spp. temperate pasture grasses

**DOI:** 10.1371/journal.pone.0249636

**Published:** 2021-04-08

**Authors:** Yuan Fu, Ann Thomas, Dagmara Gasior, John Harper, Alan Gay, Charlotte Jones, Matthew Hegarty, Torben Asp, Albert Fradera-Sola, Ian Armstead, Narcis Fernandez-Fuentes

**Affiliations:** 1 Institute of Biological, Environmental and Rural Sciences, Aberystwyth University, Aberystwyth, Ceredigion, United Kingdom; 2 Center for Quantitative Genetics and Genomics, Aarhus University, Flakkerberg, Slagelse, Denmark; 3 Quantitative Proteomics, Institute of Molecular Biology (IMB), Mainz, Germany; Universidade de Lisboa Instituto Superior de Agronomia, PORTUGAL

## Abstract

Ryegrasses *(Lolium* spp.) and fescues (*Festuca* spp.) are closely related and widely cultivated perennial forage grasses. As such, resilience in the face of abiotic stresses is an important component of their traits. We have compared patterns of differentially expressed genes (DEGs) in roots and leaves of two perennial ryegrass genotypes and a single genotype of each of a festulolium (predominantly Italian ryegrass) and meadow fescue with the onset of water stress, focussing on overall patterns of DEGs and gene ontology terms (GOs) shared by all four genotypes. Plants were established in a growing medium of vermiculite watered with nutrient solution. Leaf and root material were sampled at 35% (saturation) and, as the medium dried, at 15%, 5% and 1% estimated water contents (EWCs) and RNA extracted. Differential gene expression was evaluated comparing the EWC sampling points from RNAseq data using a combination of analysis methods. For all genotypes, the greatest numbers of DEGs were identified in the 35/1 and 5/1 comparisons in both leaves and roots. In total, 566 leaf and 643 root DEGs were common to all 4 genotypes, though a third of these leaf DEGs were not regulated in the same up/down direction in all 4 genotypes. For roots, the equivalent figure was 1% of the DEGs. GO terms shared by all four genotypes were often enriched by both up- and down-regulated DEGs in the leaf, whereas generally, only by either up- or down-regulated DEGs in the root. Overall, up-regulated leaf DEGs tended to be more genotype-specific than down-regulated leaf DEGs or root DEGs and were also associated with fewer GOs. On average, only 5–15% of the DEGs enriching common GO terms were shared by all 4 genotypes, suggesting considerable variation in DEGs between related genotypes in enacting similar biological processes.

## Introduction

Perennial pasture grasses such as *Lolium perenne* L., *L*. *multiflorum* Lam. and *Festuca pratensis* Huds. (perennial ryegrass, Italian ryegrass and meadow fescue, respectively) are exposed to a wide range of fluctuating, and not always predictable, environmental conditions. In addition, due to the nature of the agricultural systems in which they are grown, they are established as mixed populations of genotypes and species grown in swards and so inter-plant competition, both with sown and invasive plants, plays a significant role in sward development over time. Thus, in order for the perennial pasture to reflect the sown component of the sward over a number of years, pasture grasses need to exhibit sufficient phenotypic and competitive resilience to persist within these contexts [1, 2]. The necessary plasticity of response is underpinned by the gene complement of the genome and by the matching of appropriate biological responses to existing and future conditions–mediated by gene expression.

In order to understand how this resilience is manifested within temperate pasture grasses, a number of studies have looked at the contribution of differential gene expression, usually in leaves and sometimes in roots, in response to various aspects of stress within *Lolium* and *Festuca* spp. These studies have involved stresses such as cold-acclimation [3], xenobiotics [4, 5], disease resistance [6], submergence [7], salinity [8, 9], heavy metals [10, 11], as well as water-stress [12–20]. Because of the resources required to conduct and analyse transcriptomics experiments, a common approach is to compare gene expression in pairs of tolerant and susceptible genotypes and then to frame observed differences in terms of how expression profiles may be contributing to the differing responses to stress—and this can provide valuable insights. The challenge for all crop scientists and breeders, however, and particularly for those working on perennial crop species cultivated over a wide environmental range, such as perennial forage grasses, is that the components of drought tolerance may vary according to climate, season, plant age and genetic background. Thus, selection for stress tolerance in breeding programmes has to reflect the complexity of these quantitative traits [21, 22] and cannot just be focussed on candidate genes, especially as the effects of specific candidate genes may show considerable variation depending on genotypic backgrounds and genotype x environment interactions [23]. A second major challenge is, of course, the inaccessibility of the roots, the plant organs which often interface with abiotic and biotic challenges directly and in which measuring changes in gene expression, or any other physiological parameters, presents considerable difficulties [24].

In the present study, rather than focussing on the identification of candidate genes which may distinguish genotype trait performance, we have been interested in looking at the profiles of differential gene expression and associated gene ontologies which are common to 4 diverse ryegrass and fescue genotypes. These consisted of 1) a *F*. *pratensis* genotype originally sourced from a seed accession of a Russian landrace and known to be compatible with *L*. *perenne* in terms of cross-fertility and genome introgression [25]; 2) a *L*. *multiflorum* (festulolium) genotype containing a single terminal introgression on chromosome 3 derived from *F*. *arundinacea* (tall fescue); this genotype performed well in terms of survival and re-growth during drought tests and is considered to be an advanced breeding line [26]; 3) a *L*. *perenne* genotype from a Bulgarian seed accession which was growing in thin soil between cobbles in a monastery precinct and so subject to both drying and human trampling; 4) a *L*. *perenne* genotype from a semi-natural Romanian collection taken from a cattle-grazed pasture. These latter two genotypes were chosen as representatives of less well studied *L*. *perenne* germplasm from distinct gene pools and growing environments from south-eastern Europe [27] which were not likely to have been the product of intensive breeding.

The rationale for this approach was that the identification of commonalities in the abiotic stress response in a diverse set of genotypes may indicate routes for manipulating traits in a more predictable fashion. Currently, the degree to which the same abiotic stress-associated biological processes are enacted by similar gene sets is unknown for these grasses and, while any four genotypes will be a very limited subset of the available germplasm resources, it was hoped that the results would be informative in terms of evaluating this important area. To this end we have: a) compared the profiles of differentially expressed genes (DEGs) in leaves and roots in these 4 diverse *Lolium/Festuca* spp. genotypes sampled with increasing water stress; b) identified, through the framework of gene ontologies (GOs), common biological processes enacted during the progression of the stress; and c) examined the degree to which the same DEGs across species enriching the same GO terms. Through this, we have investigated the extent to which abiotic gene expression responses are conserved across these different germplasm selections.

## Materials and methods

### Experimental overview

Four diverse ryegrass/fescue genotypes were established under controlled growth room conditions in vermiculite watered with nutrient solution. Leaves and roots were sampled in quadruplicate at 35% estimated growing medium water content (EWC) and subsequently as watering was withdrawn at 15%, 5% and 1% EWC. The indication that the plants were increasingly water stressed was monitored through changes in relative water content (RWC) and leaf water conductance (LWC). RNA was extracted from leaf and root samples and RNAseq data generated on all replicates independently for every sampling point. Differential gene expression between and across sampling points was evaluated incorporating the four replicates using DESeq2, edgeR and limma-voom and requiring significant differential expression according to all three analysis programmes. Subsequent analyses of DEGs and gene ontologies were generated using the datasets from all the sampling point comparisons, both individually and combined.

### Plant material

Four *Lolium* and *Festuca* spp. genotypes were used in this study. These consisted of 1) *L*. *perenne* Ba12020/1 (Ba12), 2) *L*. *perenne* Ba9971/1 (Ba99), 3) *F*. *pratensis* Bf1183/1 (Bf11), and 4) *L*. *multiflorum* x *F*. *arundinacea* (festulolium; predominantly *L*. *multiflorum* with some retained *F*. *arundinacea*) p194/208/19 (p194). All genotypes form part of the genetic resources maintained at Aberystwyth University and their origins have been described previously (Ba12 and Ba99, [27]; Bf11, [25]; p194, [26].)

#### Experimental conditions and drought treatments

All drought treatments and subsequent physiological measurements were carried out on four replicates of each genotype at each sampling point. Clonal replicates of the four study genotypes where taken from compost grown plants maintained at ambient temperature in glasshouse conditions, post-flowering and prior to any further period of vernalisation. They were established in 6-inch pots in compost in a 20°C growth room with an 8-hour photoperiod, a light intensity of 500 μmol m^-2^ s^-1^ photosynthetic active radiation and 74% relative humidity. All subsequent experimental evaluations were carried out under these conditions. These species require long days in order to induce flowering and these short-day conditions maintained the replicates in the vegetative growth stage (i.e. no induction to flowering before or during the experimental period).

Sixteen single tillers were taken from the compost grown clonal replicates of each genotype, rinsed of compost, and transferred to containers of water until they showed new root growth, after about 6 days on average. At this point they were transferred to 90mm pots containing vermiculite (graded for horticultural use, 2–5 mm) in randomised positions to establish and were watered with a Hoagland’s solution [28] twice a week. Once established, between 15 and 21 days after tillering, watering was stopped and water content was estimated (estimated growing medium water content; EWC) using a moisture meter HH2 Delta-T meter (AT Delta-T devices, Cambridge, UK). At each estimation 3 different moisture readings were taken and averaged. Leaves and roots were sampled at 35% (full watering, day 0) and when 15%, 5% and 1% EWC levels were reached (a maximum of 13 days from start to finish). Leaf samples were cut and flash frozen in liquid nitrogen and stored at -80°C. The roots were briefly washed with distilled water to remove the growing medium and then blotted dry prior to freezing in liquid nitrogen and storage at -80°C. Four different clones were sampled at each EWC point, deriving 4 biological replicates for each stage.

#### Leaf relative water content

Estimations of leaf relative water content (RWC) were measured at 35%, 15%, 5% and 1% EWC. RWC estimations were carried out on additional replicates of the same genotypes, prepared and grown identically to the plants used for RNA extraction, as follows. Three leaves from each replicate were removed, a 5cm mid-section was cut from each leaf, and the fresh weight (FW) was measured. This excised section was then placed in a sterile tube containing 3ml water, capped and left at 4°C for 24 hours. After this period, the leaf sections were blotted and turgid weight (TW) was measured. The sections were then dried for 24 hours at 80°C for the dry weight (DW). RWC was calculated as (FW-DW/TW-DW) x 100.

#### Leaf water conductance

Leaf water conductance (LWC) was measured using an AP4 Porometer (Delta T Devices, Cambridge, UK). Measurements were taken c. 3 hours after the start of the light period on the adaxial side of the top-most fully expanded leaf of 5 separate shoots from each of three replicates per genotype. The values reported for each time point are averages of the 15 measurements on each genotype.

### RNA extraction and sequencing

Total RNA was extracted from leaf material using the Trizol method (Sigma-Aldrich, Poole, UK) and quantified using Qubit fluorescence spectrophotometry. 1ug of total RNA was used per sample for library construction according to the Illumina TruSeq Stranded mRNA Library Preparation Kit protocol. Samples were indexed such that 24 samples could be multiplexed per lane of a HiSeq2500 platform (2x126bp format). Samples were run across two high-output flowcells and reads demultiplexed using the bcl2fastq script.

### RNAseq processing: Quality control and mapping

Raw reads were processed using Trimmomatic v.0.33 [29] to remove adapters with following parameters (optimized after several run tests): ILLUMINACLIP:TruSeq3-PE-2.fa LEADING:15 SLIDINGWINDOW:4:15 MINLEN:30 HEADCROP:12, and the quality of resulting trimmed and clean reads assessed using FastQC v.0.11 [30]. Reads were then mapped to the reference diploid genome of *L*. *perenn*e homozygous genotype p226/135/16 [31] using the splice-aware mapper Hisat2 v.2.0.0 [32] with default parameters and the special options—*phred33* (required to handle Illumina reads) and—*dta* (required downstream processing with StringTie). Subsequently transcripts were assembled and merged using StringTie v1.1.0 [33] using default parameters. The completeness of each transcriptome was assessed using BUSCO [34] on the *early_release plantdb* dataset, composed of 1440 core genes.

### Differentially expressed genes

Prior to calling of DEGs a quality control based on principal component analysis (PCA) and read number was carried out to select the best parameters for calling DEGs.

Raw counts of transcripts were retrieved using an R script from precomputed mapping files (bam files). Derived counts were used as inputs to call DEGs using edgeR [35], DESeq2 [36] and limma-voom [37, 38], across the 4 EWC sampling points. Comparisons were made as follows. 1) Against Reference (AR), with differential expression being evaluated by comparing the 35% EWC sampling point with all other sampling points (35/15, 35/5 and 35/1) and 2) Time Course (TC), with differential expression being evaluated between consecutive sampling points (35/15, 15/5 and 5/1). The 35/15 comparison was, therefore, the same for both AR and TC. All comparisons were carried out for all 4 genotypes independently in both leaf and root tissues. Transcripts were considered to be differentially expressed when cut-off threshold criteria of log_2_ fold change (LFC) >1.2 and a false discovery rate (FDR) of ≤ 0.01 (1%) were met by all three analysis programmes.

#### Terms used to describe patterns of expression of DEGs

For each genotype individually, DEGs that were significantly up-regulated at one or more comparison stage(s) and not significantly down-regulated for the remaining comparison stages(s) are referred to as u-DEGs. DEGs that were significantly down-regulated at one or more comparison stage(s) and not significantly up-regulated for the remaining comparison stages(s) are referred to as d-DEGs. Individual DEGs that showed both significant up- and down-regulation at different comparison stages within the same genotype are referred to as i-DEGs (inconsistent-DEGs). When referring to the same DEG(s) in more than one genotype and the differential regulation varied across genotypes in terms of direction, these are referred to as m-DEGs (mixed-DEGs).

Core DEGs: the set of genes which were differentially regulated in all the genotypes for at least one of the AR or TC comparison stages (d-DEGs, u-DEGS or m-DEGs).

Trend DEGs: for each genotype individually, the set of genes that were differentially regulated for at least one of the AR or TC comparison stages (d-DEGs, u-DEGS or i-DEGs).

Expression categories: for each genotype, individual genes within the trend set were also described using a 3-letter code indicating their direction of differential expression at each of the EWC comparisons. The first, second and third letter indicate the direction of expression at the AR 35/15, 35/5 and 35/1 or the TC 35/15, 15/5 and 5/1 comparisons; *n* = not significant; *u* = up-regulated; *d* = down-regulated. Thus, an AR DEG described as *ndd* was non-significant for the 35/15 comparison but significantly down regulated for the 35/5 and 35/1 comparisons. A TC DEG described as *unn* was significantly up-regulated for the 35/15 comparison but was non-significant at the 15/5 and 5/1 comparisons.

### Functional annotation of DEGs and GO term enrichment

The reference genome was functionally re-annotated using OmicsBox [39] as a prior step before computing gene ontology (GO) term enrichments. The functional annotation was carried out as follows: CloudBlast (blastx-fast) searches were performed on the nr (v5) database, restricted to Viridiplantae, with default parameters. InterPro searches were performed using InterProScan (CloudIPS) [40] on all the families, domains, sites and repeats (S1 Table).

The significance of GO term enrichments was estimated using Fisher’s exact test with a false discovery rate (FDR) ≤5% using OmicsBox. Associated KEGG [41] enzyme activity codes were also identified using OmicsBox. Significantly enriched GO terms (referred to as GOs from this point forward) were generated with 3 different methods of compiling the DEGs, using the core sets, the trend sets and using the 3-letter expression categories. For both leaves and roots, GOs from these sets which could be connected through hierarchies visualised using EBI QuickGO [42] were grouped according to a putative common functional area.

### Statistical procedures

One-way analysis of variance, Tukey-Kramer, χ^2^ and FDRs relating to these, and Bartlett’s test for homogeneity of variances were carried out using tools provided in [43] using untransformed datasets with thresholds of p≤0.05 and FDR controlled at 5%. Adjusted probability values for determining DEGs were derived directly from each individual program, i.e. DESeq2, edgeR and limma-voom, incorporating the variation across the 4 biological replicates as described above. Significant enrichments of GO terms were identified through the OmicsBox software using Fisher’s Exact Test (p ≤0.05) and the FDR controlled at 5%.

## Results

### Pre-processing, mapping, and quality of sequencing across replicates; KEGG enzyme activities and pathways

Details of pre-processing, mapping, and quality of sequencing across replicates and KEGG enzyme activities associated with DEGs are provided in S1 Results.

### Summary of DEGs identified

Over the 3 AR comparisons an average of 2717 and 3040 DEGs were detected per genotype in both leaf and root; the equivalent figures for the TC comparisons were lower at 1599 DEGS for leaf and 1142 DEGs for root (Table 1). However, there were differences between genotypes in terms of the overall numbers with noticeably more DEGs being detected for genotypes Ba12 and p194 for both AR and TC in comparison to Ba99 and Bf11. This was particularly marked for the TC comparison in the root tissue, with only 20 DEGs identified for the TC comparison in Bf11, as compared to 2297 DEGs for Ba12. The large majority of the DEGs were either u-DEGs or d-DEGs, with u-DEGs and d-DEGs being c. evenly represented. Very few DEGS were regulated in one direction at one comparison point and then in the opposite direction at a subsequent comparison point (i-DEGs) for both AR leaf and root comparisons–though slightly higher numbers were detected for the TC comparisons. There was also a slight difference between leaves and roots in terms of the total number of i-DEGs, with AR i-DEGs representing c. 0.9% of the total for leaves and 0.07% for the roots. For the TC comparisons the equivalent comparisons were 12% and 2% respectively.

**Table 1:** The numbers of up (u-), down (d-) or inconsistent (i-) differentially expressed genes (DEGs) in the leaf and root of the 4 genotypes according to comparison type.

^1^Comparison	Tissue	Direction	Ba12	Ba99	Bf11	p194	Average
Across AR and TC	leaf	Any	3330	2687	2084	3844	2986
	root	Any	4607	2225	2218	3488	3135
AR	leaf	d-DEGs	1737	1418	953	1966	1519
		u-DEGs	1114	1197	1089	1298	1175
		i-DEGs	64	2	10	18	24
		Total	2915	2617	2052	3282	2717
	root	d-DEGs	2133	1220	987	1703	1493
		u-DEGs	2205	998	1231	1676	1500
		i-DEGs	3	0	0	5	2
		Total	4341	2218	2218	3384	3040
TC	leaf	d-DEGs	831	319	448	1380	745
		u-DEGs	888	253	565	968	669
		i-DEGs	227	11	150	356	186
		Total	1946	583	1163	2704	1599
	root	d-DEGs	936	182	7	874	498
		u-DEGs	1338	244	13	873	613
		i-DEGs	23	0	0	78	25
		Total	2297	426	20	1825	1142

^1^Comparisons are against reference (AR) and time course (TC). The totals in the AR and TC rows exceed those in the Across AR and TC rows due to some DEGs being significant for both AR and TC comparisons

### Patterns of differential gene expression with increasing water stress

**Fig 1:**
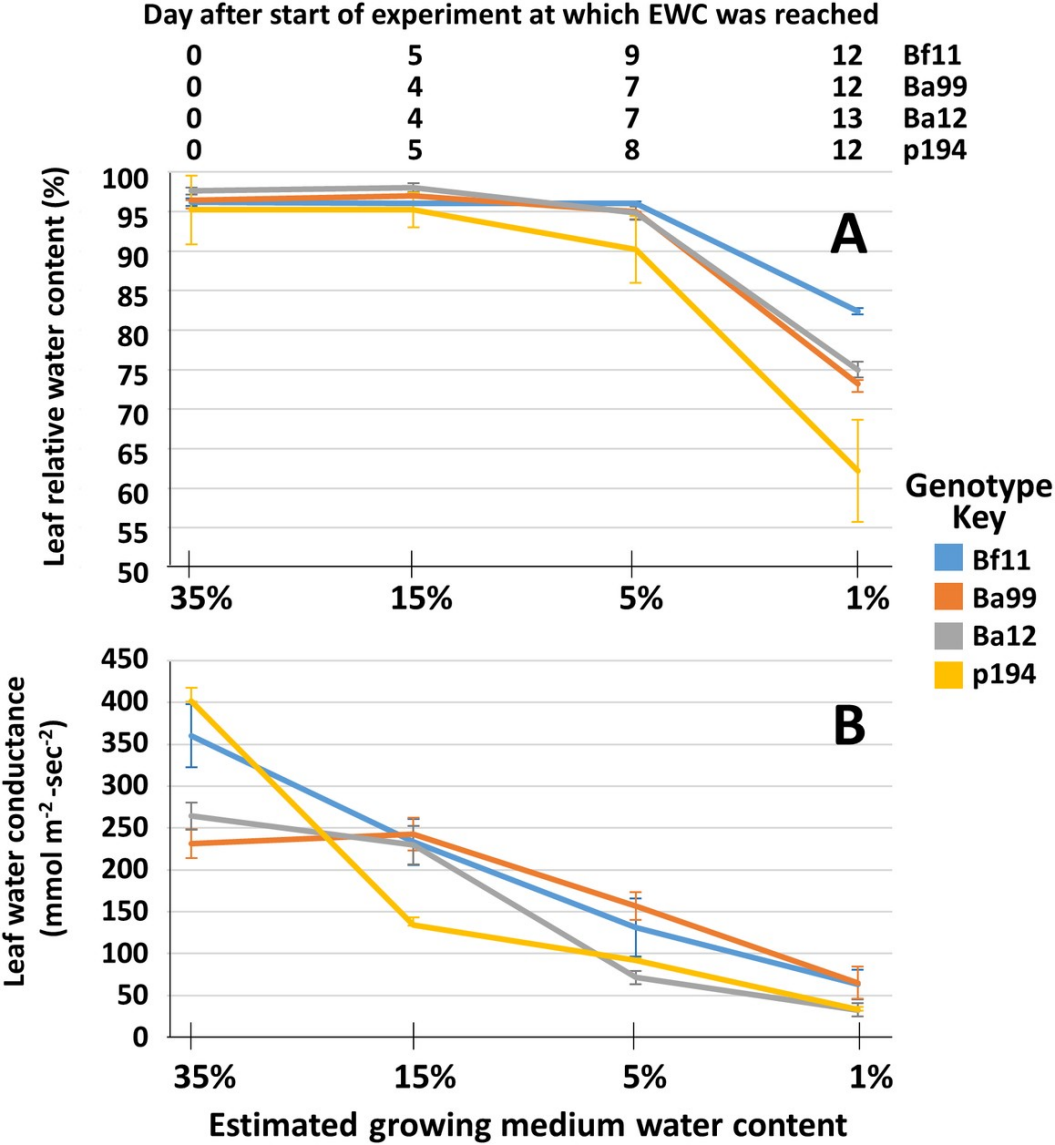
Indicators of increasing water stress with sampling points. (A) Leaf relative water content and (B) leaf water conductance for the 4 genotypes at the estimated growing medium water content (EWC) sampling points. Error bars represent the standard errors across the 4 replicates at each EWC sampling point. The days after the start of the experiment at which the EWC sampling point was reached is given above panel A.

Measurements of RWC and LWC indicated the initial stages of physiological response to water stress over a period of 12 to 13 days after the withdrawal of watering (Fig 1) and for the purposes of this study, these measurements indicated that all the genotypes were increasingly water stressed as the EWC decreased. The experiment was not designed specifically for the purposes of evaluating RWC and LWC traits between genotypes and so no statistical comparisons were made to evaluate inter-genotype differences. The number of days that passed before the genotypes reached the given EWC were as follows: 35% EWC, day 0, all genotypes; 15% EWC, day 4 –Ba99 and Ba12, day 5 –Bf11 and p194; 5% EWC, day 7 –Ba99 and Ba12, day 8—p194, day 9 –Bf11; 1% EWC, day 12 –Bf11, Ba99 and p194, day 13, p194.

**Fig 2:**
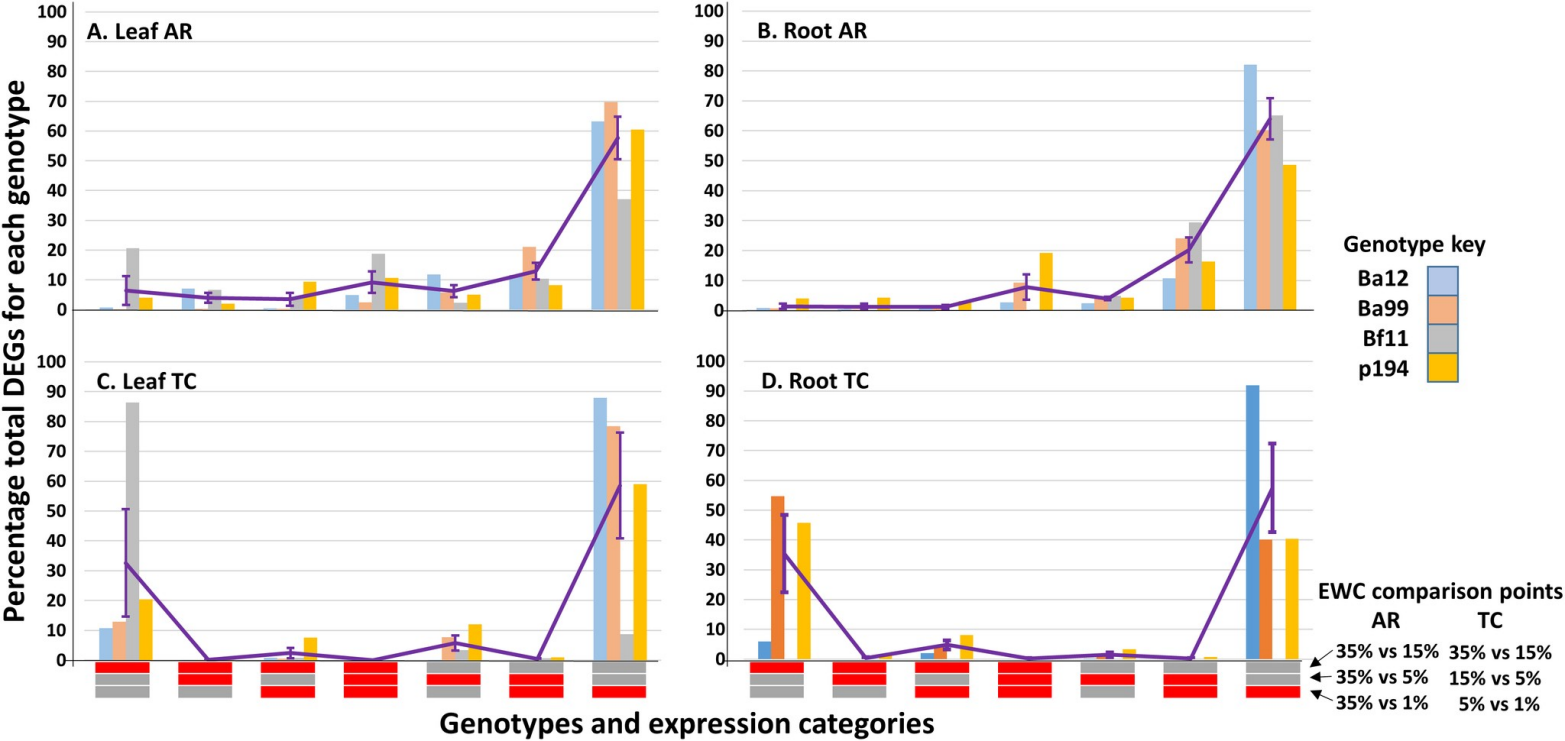
Relative proportions of differentially expressed genes (DEGs) across genotypes and expression categories. **A.** leaf against reference (AR); **B.** root AR; **C.** leaf time course (TC); **D.** root TC. For the root TC DEGs, Bf11 has been omitted as only 20 DEGs of any kind were present in this class. The purple line connects the average proportion of DEGs across genotypes for each expression category. Error bars indicates standard errors. The expression categories are indicated as horizontal bars beneath the x axis where red means genes differentially expressed and grey not differentially expressed for that component of the expression category, respectively. Estimated growing medium water contents (EWC) associated with the expression category components are given at the end of the x axis.

The respective distributions of DEGs over these EWC comparisons were uneven for both AR and TC comparisons but relatively consistent across genotypes, particularly for AR comparisons (illustrated as percentage proportions in Fig 2 with full details for all DEGs supplied in S4 Table). In terms of overall trends, for AR leaf and root comparisons, the *nnd/nnu* expression categories contained the majority of DEGs, though with some genotype-dependent differences, particularly for Bf11 which had higher proportions of DEGs present in the *dnn/unn* and *ddd/uuu* leaf expression categories compared to the other 3 genotypes (and, consequently, lower proportions in the *nnd/nnu* expression categories). For the TC comparisons for both leaves and roots the majority of the DEGs were identified either in the *dnn/unn* or the *nnd/nnu* expression categories, though, again with inter-genotype differences. For Bf11, the majority of the TC leaf DEGs were present in the *dnn/unn* expression categories whereas for the other 3 genotypes the majority of the DEGs were in the *nnd/nnu* expression categories. For the root TC, both Ba99 and P194 had c. even proportions of DEGs distributed between the *dnn/unn* and *nnd/nnu* expression categories, whereas the large majority of DEGs associated with Ba12 were in the *nnd/nnu* expression category alone.

### Core DEGs, leaves and roots

**Fig 3:**
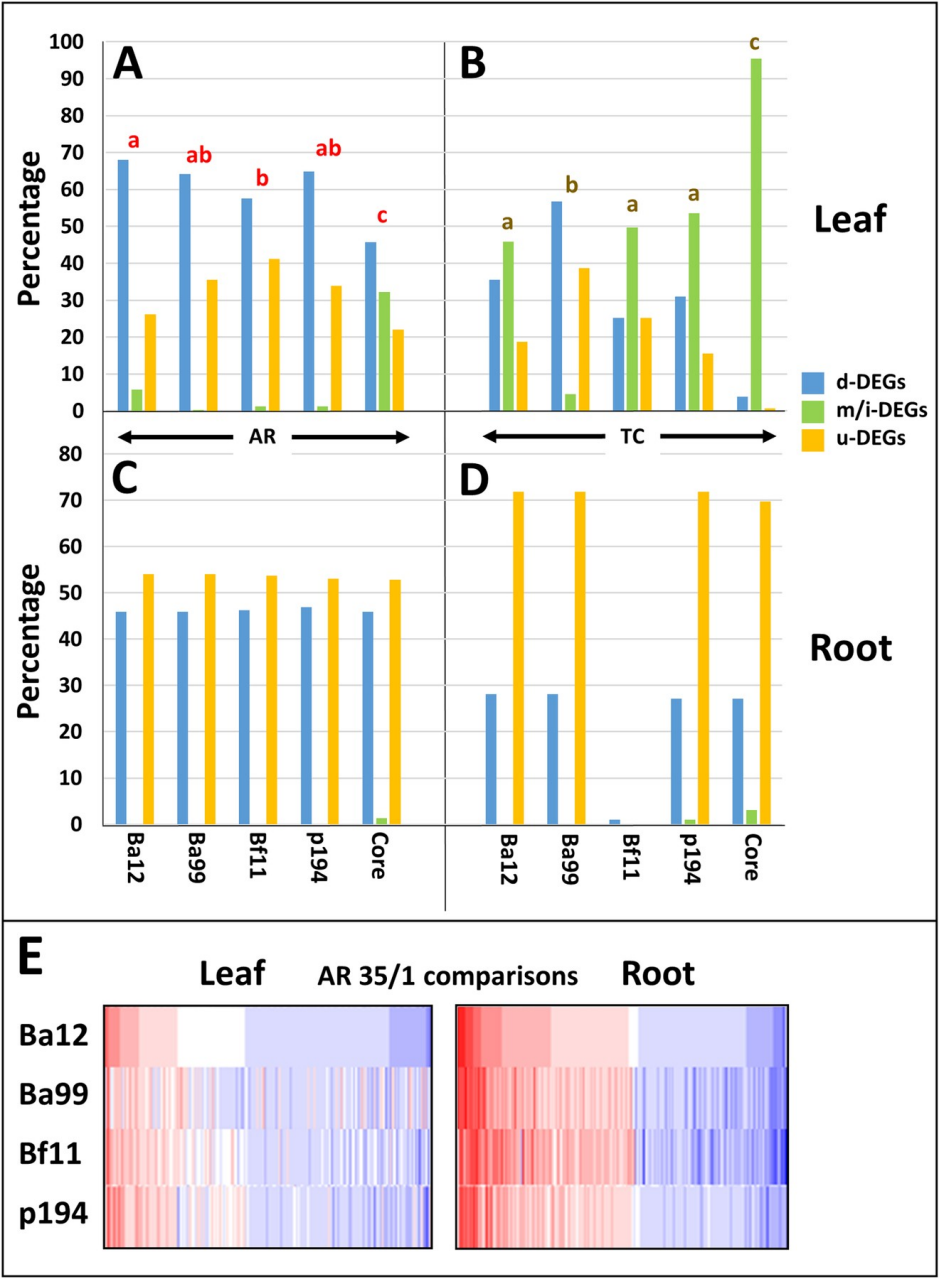
The patterns of expression of core differentially expressed genes according to genotype and comparison. **A—D.** The patterns of up (u-), down (d-) mixed (m-) or inconsistent (i-) differentially expressed genes (DEGs) in the leaf and root for core DEGs and the same DEGs taken from the Trend data sets. m-DEGs refers to the core set and i-DEGs refers to the trend sets. Comparisons were made between genotypes within: **A.** leaf against reference (AR) core DEGs: **B.** leaf time course (TC) core DEGs; **C.** root AR core DEGs; **D.** root TC core DEGs. The relative proportions of the 3 classes were compared across genotypes and the core set in leaves and root using χ^2^. Where the initial χ^2^ was significant (p < 0.05), post hoc pairwise genotype and core χ^2^ comparisons which were significantly different from each other, using a 5% FDR to control for multiple testing, do not share any of the same red (AR) or brown (TC) letters above the categories. No significant differences were found for the root. **E.** Heat maps showing the patterns of up- and down-regulation of the core DEG set for leaves and roots at the 35% vs 1% (35/1) estimated growing medium water content comparison. Both leaf and root sets were ordered from maximum to minimum in terms of log fold change (LFC; dark red to dark blue) according to the Ba12 LFC values. It can be seen that patterns of up- and down-regulation are more strongly conserved in the roots as compared to the leaves.

Using the core DEG criteria identified a total of 566 DEGs from the leaf and 643 DEGs from the root, of which 69 DEGs were in common to both leaf and root. One observation concerning this core set was that, while they were mostly u-DEGs or d-DEGs for the individual genotypes within the AR leaf and root comparisons (*i*.*e*. very few of them were i-DEGs), the same DEG was quite frequently regulated in different directions across all the genotypes (m-DEGs) within the leaf, but rarely within the root. This is illustrated in Fig 3 in comparison with the direction of regulation of the core genes but described according to the genotype-specific trend criteria. For example, an average of 2% of the leaf AR DEGs were classified as i-DEGs on an individual genotype basis, whereas m-DEGs accounted for 32% of the total core set (p < 0.05; Fig 3). In contrast, for root AR DEGs there were no i-DEGs on an individual genotype basis and only 1% m-DEGs across all genotypes. The TC leaf DEGs showed a similar trend to the AR leaf DEGs, though with far greater numbers, with an average of 38% of the DEGs classified as i-DEGs on an individual genotype basis and 95% as m-DEGs across all genotypes (p < 0.05; Fig 3). For the TC root DEGs, as with the AR root DEGs the number of i-DEGs was >1% on an individual genotype basis and m-DEGs c. 3% across all genotypes. Thus, the direction of regulation of comparable (core) DEGs was much more highly conserved across all 4 genotypes in the root than in the leaf.

### Identification of GO terms associated with DEGs

In this study, we have focussed only on those GOs which were identified either in the core set or for all four genotypes independently in either or both of the trend or expression category sets. The putative common functional areas connecting GOs are given in Tables 2 and 3. The complete list of GO terms as identified for core, trend and 3-letter code datasets are described in S5 and S6 Tables.

**Table 2:** The number of significantly enriched GO terms and total numbers of associated DEGs assigned to putative functional areas in the leaf for the core and trend datasets.

		CORE				Ba12 TREND				Ba99 TREND				Bf11 TREND				p194 TREND			
		GOs^2^			DEGs^3^	GOs			DEGs	GOs			DEGs	GOs			DEGs	GOs			DEGs
Leaf functional GO groups^1^		D	U	M		D	U	I		D	U	I		D	U	I		D	U	I	
Cell periphery	CC	5	0	4	192	9	7	3	1238	9	7	0	1069	9	7	0	795	7	7	0	1329
Nucleotide binding	MF	0	0	0	0	12	4	0	886	6	12	0	805	12	12	0	610	12	12	1	1215
Carbohydrate metabolism	BP	3	1	14	174	19	7	1	832	17	7	0	989	17	14	0	743	18	13	5	1272
Photosynthesis	CC	28	1	3	204	29	0	0	620	29	0	0	559	29	3	0	674	27	1	0	1144
Protein phosphorylation	BP	1	0	0	10	12	4	0	620	5	9	0	522	12	4	0	405	12	0	0	558
Organelle	CC	7	0	3	197	6	0	0	609	6	0	0	552	6	5	0	670	6	3	1	1244
Response to stimulus	BP	7	0	5	89	25	22	3	535	24	14	0	409	20	23	0	322	29	20	2	575
Transcription	BP	0	0	18	41	19	4	17	529	1	4	0	247	5	17	0	324	11	14	19	643
Metal ion binding	MF	0	0	0	0	4	5	2	498	3	5	0	417	3	5	0	303	3	4	1	546
Kinase activity	MF	0	0	1	4	6	5	0	452	0	6	0	233	5	6	0	302	6	1	0	389
Transmembrane transport	BP	0	0	5	31	22	16	16	387	16	25	0	319	23	11	0	265	22	23	6	474
Oxidoreducase activity	MF	2	0	0	30	3	5	2	353	2	5	0	321	2	5	0	215	1	5	0	337
Amino acid metabolism	BP	4	0	0	16	11	8	0	346	12	10	0	336	13	5	0	235	12	9	0	443
Transporter activity	MF	0	0	3	24	22	12	10	286	13	23	0	228	24	10	0	190	22	20	1	331
Photosynthesis	BP	27	0	1	78	34	0	0	268	36	1	0	306	34	0	0	192	33	0	0	308
Photosynthesis	MF	4	0	0	27	5	2	0	133	5	2	0	145	5	3	0	133	4	2	0	147
Transcription	MF	0	0	2	15	2	1	2	116	0	2	0	43	0	2	0	44	2	2	2	133
Glycosyl transferase	MF	0	1	0	8	5	3	0	115	5	2	0	104	0	5	0	51	5	5	0	131
DNA binding	MF	0	0	1	13	1	2	1	108	0	1	0	37	0	1	0	42	1	1	1	84
Carbohydrate lyase activity	MF	2	1	3	30	2	3	0	101	2	3	0	95	3	1	0	58	2	3	0	110
Phosphatase activity	MF	0	1	0	8	4	0	0	97	4	0	0	72	4	3	0	125	4	0	0	106
Extracellular	CC	2	0	0	16	2	0	0	75	2	0	0	61	2	0	0	47	2	0	0	71
Nucleotide metabolism	BP	4	0	0	13	7	0	0	71	7	0	0	60	6	0	0	32	7	0	0	81
Signalling	BP	0	0	0	0	4	4	0	71	4	3	0	148	1	4	0	84	4	4	0	225
Lipid metabolic process	BP	0	0	0	0	2	6	2	71	3	6	0	166	0	6	0	66	5	3	0	200
Organic acid metabolism	BP	0	0	4	9	0	3	0	24	0	3	0	23	0	6	0	22	0	3	0	35
Phenylpropanoid metabolism	BP	0	0	0	0	0	2	1	21	0	0	0	0	0	4	0	11	0	2	2	23
NADP	BP	0	0	0	0	2	0	0	14	2	0	0	11	2	0	0	10	2	0	0	15
Response to light	BP	0	0	4	10	2	0	0	11	2	2	0	14	0	0	0	0	2	0	2	16
Translation	BP	4	0	0	23	0	0	0	0	0	0	0	0	3	0	0	50	0	0	0	0

^1^CC: Cellular Component; MF: Molecular Function; BP: Biological Process

^2^D, U, M and I refer to GO terms enriched by DEGs showing up, down, mixed or inconsistent regulation, respectively.

^3^DEGs the total number of DEGs contributing to the enrichment of the GO terms within that functional group

**Table 3:** The number of significantly enriched GO terms and total numbers of associated DEGs assigned to putative functional areas in the root for the core and trend datasets.

		CORE				Ba12 TREND				Ba99 TREND				Bf11 TREND				p194 TREND			
		GOs^2^			DEGs^3^	GOs			DEGs	GOs			DEGs	GOs			DEGs	GOs			DEGs
Root functional GO groups^1^		D	U	M		D	U	I		D	U	I		D	U	I		D	U	I	
Cell periphery	CC	2	7	0	194	8	7	0	1573	7	9	0	772	6	9	0	833	9	7	0	1272
Ion binding	MF	5	2	0	141	4	6	0	1257	5	4	0	710	5	5	0	726	6	5	0	1050
Nucleotide binding	MF	14	1	0	129	16	1	0	813	16	1	0	493	16	14	0	761	16	3	0	1032
Organelle	CC	2	0	0	32	6	0	0	800	2	0	0	103	6	0	0	378	0	1	0	557
Protein phosphorylation	BP	5	0	0	66	8	0	0	786	8	0	0	401	8	4	0	523	8	0	0	558
Carbohydrate metabolism	BP	3	1	0	118	16	0	0	710	3	9	0	145	16	1	0	401	14	5	0	630
Response to chemical stress	BP	19	3	0	105	20	4	0	691	24	6	0	368	25	5	0	416	25	5	1	570
Transmembrane transport	BP	1	27	0	78	4	27	0	546	4	26	0	306	3	27	0	246	1	26	0	365
Phosphotransferase activity	MF	7	0	0	64	7	0	0	498	7	0	0	203	8	6	0	489	7	1	0	666
Amino acid metabolism	BP	22	0	0	47	11	0	0	483	14	0	0	286	14	6	0	394	17	0	0	412
Oxidoreductase activity	MF	1	4	0	69	1	6	0	439	2	5	0	230	1	5	0	254	2	5	0	409
Transmembrane transporter activity	MF	0	27	0	62	0	29	0	266	2	28	0	243	2	29	0	210	2	28	6	328
Response to stimulus	BP	0	13	0	35	1	20	0	236	0	20	0	95	2	20	0	175	3	20	1	239
Cell wall and extracellular region	CC	4	0	0	28	4	0	0	182	4	1	0	120	4	0	0	86	4	1	0	205
heme binding	MF	2	0	0	13	2	2	0	147	2	2	0	87	2	2	0	100	2	2	0	154
Cytoskeleton-MF	MF	12	0	0	27	12	0	0	145	12	0	0	77	12	0	0	81	7	0	0	42
Transferase activity	MF	0	3	0	19	2	5	0	140	0	5	0	42	0	5	0	61	3	4	0	103
DNA binding	MF	0	5	0	18	1	2	0	127	0	0	0	0	0	5	0	53	0	3	0	23
Cell cycle	BP	3	0	0	16	4	0	0	110	4	0	0	46	4	0	0	74	0	0	0	0
Cytoskeleton–CC	CC	12	0	0	20	12	0	0	109	12	0	0	54	12	0	0	72	11	0	0	47
Negative regulation of peptidase activity	BP	2	5	0	19	2	6	0	101	1	6	0	19	2	6	0	59	1	5	0	19
Glycosyl hydrolase activity	MF	0	2	0	15	2	0	0	83	2	2	0	80	2	1	0	74	2	2	0	117
Cytoskeleton–BP	BP	3	0	0	2	3	0	0	75	3	0	0	36	3	0	0	50	3	0	0	31
Antioxidant activity	MF	5	0	0	17	3	0	0	65	4	0	0	35	4	0	0	35	4	0	0	53
Cell-cell junctions	CC	5	0	0	11	5	0	0	56	5	0	0	26	5	0	0	32	5	0	0	38
Homeostasis	BP	0	11	0	14	0	8	0	47	1	11	0	36	0	11	0	33	0	2	0	35
Cellularisation	BP	2	0	0	3	0	0	0	0	0	0	0	0	2	0	0	4	0	0	0	0
Phosphoric ester hydrolase activity	MF	2	0	0	2	0	0	0	0	0	0	0	0	2	0	0	2	0	0	0	0
inositol kinase activity	MF	6	0	0	2	0	0	0	0	0	0	0	0	6	0	0	2	0	0	0	0
Alcohol dehydrogenase activity	MF	3	0	0	4	0	0	0	0	3	0	0	5	3	0	0	6	3	0	0	10
Vitamin B6 binding	MF	2	0	0	6	0	0	0	0	0	0	0	0	2	0	0	12	0	0	0	6

^1^CC: Cellular Component; MF: Molecular Function; BP: Biological Process

^2^D, U, M and I refer to GO terms enriched by DEGs showing up, down, mixed or inconsistent regulation, respectively.

^3^DEGs the total number of DEGs contributing to the enrichment of the GO terms within that functional group

There was variation between genotypes in terms of the overall patterns of appearance of DEGs and related GO terms. For example, leaf GO terms from d-DEGS associated with, particularly, response to stimulus, carbohydrate, amino acid, protein, lipid metabolism and transcription were detected at an earlier stage in Bf11 and p194 (S5 Table) using both AR and TC evaluations. In addition, Bf11 showed an indication of up-regulation of DEGs associated with these same processes, but also transmembrane transport, and organic acid and phenylpropanoid metabolism. In contrast to the leaf, the overall patterns of differential gene expression in the root were more consistent across genotypes and showed an increase across the EWC comparison points. However, p194 had a higher proportion of DEGs at the first EWC comparison point and also, generally, showed earlier down-regulation of GOs associated with response to chemical stress, amino acid and carbohydrate metabolism and cell wall and periphery associated functions (amongst others–see S6 Table). So, the overall patterns of expression varied across the genotypes as the water-stress increased and this was associated with earlier or later onsets of programmes of biological activities as indicated by GO terms.

#### DEGs enriching the same GO terms across genotypes

**Fig 4:**
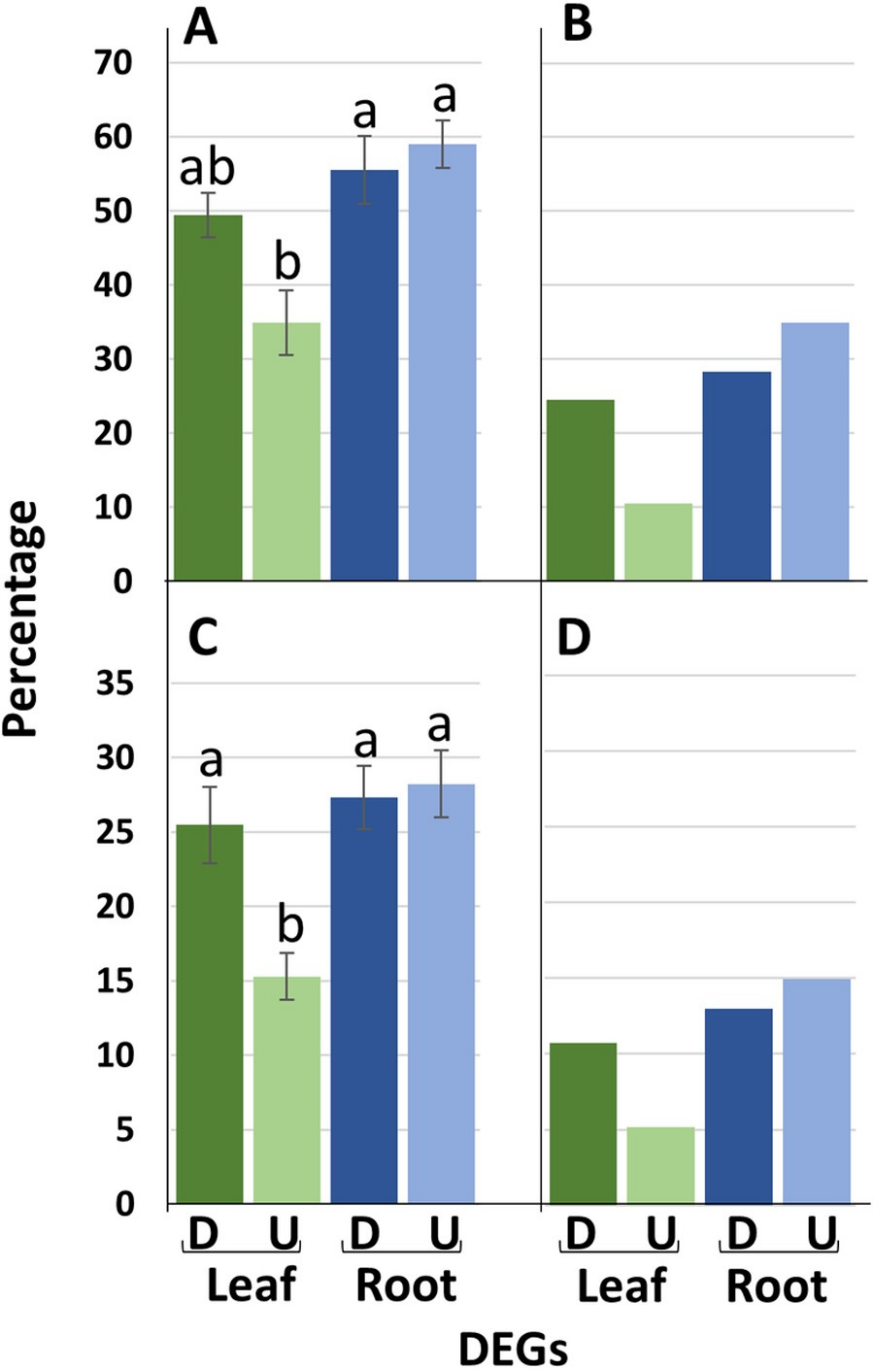
The percentage proportions of shared up-(U) and down-(D) regulated differentially expressed genes (DEGs) in leaf and root. A and B describe the distribution of just those DEGs which contribute to the significant enrichment of GO terms identified for all 4 genotypes; **A** the average percentage of the six possible pairwise genotype comparisons and **B** the percentage of DEGs present in all 4 genotypes. **C** and **D** uses the complete set of DEGs; **C** the average percentage of the six possible pairwise genotype comparisons and **D** the percentage of DEGs present in all 4 genotypes. Error bars represent standard errors; letter codes indicate significant differences (p<0.05) according to the Tukey-Kramer test following one-way analysis of variance (p<0.05). **B** and **D** are based on the single available measurements for each class.

The GOs suggest the biological programmes that were initiated in the 4 genotypes in response to water-stress. Through comparing the DEGs which contribute to the significant enrichment of the same GO terms across the 4 genotypes we can assess to what extent the same biological programmes are enacted by the same DEGs in the different genotypes. Fig 4A and 4B illustrate this for DEGs contributing to the enrichment of the trend set GOs described in S5 and S6 Tables which were common within genotype pair comparisons and also common to all 4 genotypes. The equivalent figures for DEGs within the complete trend set (i.e., irrespective of contribution to GO enrichment) are given for comparison in Fig 4C and 4D. For root u-DEGS and d-DEGs genotype pair comparisons, an average of c. 58% of the DEGs enriching GOs for one of the genotypes were also enriching GOs for the other genotype. However, this figure dropped to c. 34% for leaf u-DEGs–with leaf d-DEGs being more intermediate (p < 0.05; Fig 4A and 4C). The same trend, though lower overall numbers, was seen for the DEGs common to all 4 genotypes enriching GOs (Fig 4B), with a figure of c. 10% for the leaf u-DEGs compared to an average of c. 30% for the other 3 categories. When the GOs were grouped according to the putative functional areas described in Tables 2 and 3 (illustrated in S2 Fig) the same general trend can be seen–and so this is not specific to a particular putative function. Thus, there is an emerging pattern that leaf u-DEGs, whether associated with GOs or not, were less strongly conserved than the other groups. This can be extended to note that not only were leaf u-DEGs less well conserved across the genotypes, they were, overall, also associated with fewer GO terms (c. 38% and 62%, for leaf u-DEGs and d-DEGs, respectively) averaged across the 4 genotypes. The equivalent figures for the roots were c. 46% and 54%. (see S5 and S6 Tables).

#### Comparing leaf and root GO terms

A number of the overall groupings were similar across leaves and roots, associated with amino acid and carbohydrate metabolism, DNA and nucleotide binding, protein phosphorylation, organelle, oxidoreductase activity, transmembrane activity and response to stimulus (Tables 2 and 3). However, a striking difference between the leaves and the roots was that, with a few exceptions, such as photosynthesis and cell periphery, GO groups identified in the leaves tended to consist of GOs enriched by u-DEGs and d-DEGs in more even proportions, whereas those identified in the roots tended to consist of GOs enriched predominantly by either u-DEGs or d-DEGs. Notably, while GOs contributing to response to stimulus and transmembrane transport were enriched by mostly u-DEGs in the root, the contribution of u-DEGs and d-DEGs was more similar in the leaves (Tables 2 and 3 and, in greater detail in S5 and S6 Tables). However, two groups of GOs in the leaves were atypical in their up/down expression profiles. The first was photosynthesis which was associated with very few GOs enriched by u-DEGs. And the second group was transcription, which was unusual not because of the balance of GOs enriched for u-DEGs and d-DEGs, but because of the relatively high proportion of GOs that were also enriched for m-DEGs and i-DEGs. In the core set, a total of 33 genes contributed to the enrichment of GO:0006355 (regulation of transcription, DNA-templated) and these all showed inconsistent directions of regulation across the 4 genotypes. Similarly, from the trend data, of the 222 genes that were associated with enrichment of the same GO term and present in more than one of the genotypes, c. 40% were i-DEGs. In contrast, while there was consistent enrichment for a number of transcription factors (*i*.*e*., and so strong support for GO:0006355) in the leaf, this GO term was not strongly supported in the root.

A more focussed set of transcription-related GO terms were enriched by u-DEGs in both leaf and root. These were GO:0043620 (regulation of DNA-templated transcription in response to stress), GO:0043618 (regulation of transcription from RNA polymerase II promoter in response to stress), GO:0036003 (positive regulation of transcription from RNA polymerase II promoter in response to stress) and GO:0061408 (positive regulation of transcription from RNA polymerase II promoter in response to heat stress). These GO terms were included in the transcription group in the leaf but the response to stimulus group in the root (S5 and S6 Tables). In the root, these GO terms were enriched by a total of 12 u-DEGs, 9 of which were annotated as heat stress transcription factors and 5 of which were differentially expressed in all 4 genotypes. In the leaf, the GO terms were enriched by a total of 10 u-DEGs, 8 of which were annotated as heat-stress transcription factors and 7 of which were also present in the root set. None of the leaf u-DEGs were differentially expressed in all 4 genotypes (S7 Table) again indicating fewer genotype-specific differences in the root.

## Discussion

In this study we have compared the root and leaf transcriptomic responses of 4 *Lolium* and *Festuca* spp. genotypes with increasing water stress. The genotypes under study were chosen not because of pre-established differences in their performance under drought–though these may exist depending on the conditions imposed–but in order to look at the range of response generated from diverse material in a tightly defined experimental situation. Following from this, the aim was not, as in many studies, to identify candidate genes directly in drought-tolerant and drought-susceptible lines or, more generally, to compare transcriptomes from stressed and unstressed genotypes, but to begin to develop a greater understanding of common gene expression responses of these temperate grasses under stress conditions. The rationale for this was that, while it is certain that stress response quantitative trait loci (QTL) can be detected in defined populations [44–50] transferring such QTL into breeding populations is often a considerable and long-term challenge–presumably because gene effects (even major gene effects) can be very background dependent [23]. Thus, in future work, it is hoped that we may be able to evaluate whether a desired QTL effect (e.g. for drought tolerance) is associated with more or less highly conserved differential gene expression. This may, in turn, enable us to predict the trait outcome of genotype combining with greater reliability and represent a route for translating molecular research into desired practical outcomes.

We were also interested in contributing to the scanter knowledge that exists on root transcriptomics for these grasses and, thus, the experimental system we chose allowed us to sample both roots and leaves over a series of assay points relating to increased drying of the growing medium. This system was used in a previous study on perennial ryegrass in which the combination of analytical approaches to DEG identification and GOs was also discussed [14]. Clearly, all experimental systems which allow for root sampling, whether hydroponic or other hydrated solid media [e.g., 13, 18, 51] are open to criticism as to the degree to which the imposed environmental conditions mimic growth under more natural situations, but it is hoped that through approaches such as the one described in this paper we can begin to build up a body of knowledge relevant to this task.

One observation of particular note was the different patterns of differential gene expression that distinguished roots and leaves, in that DEGs associated with roots in all 4 genotypes (the core set) were more likely to be regulated in the same direction for the 4 genotypes than in the leaves. While there are a number of published comparisons of root and leaf transcriptomes from the same genotypes [52–58] we are not aware that this particular aspect has been directly studied or reported previously. These overall patterns of differential gene expression were also reflected in the patterns of related GO terms, in that particular biological processes were more likely to be enriched by either u-DEGS or d-DEGs in the root, but often by both in the leaves. Whether this reflects that control of the biological programmes initiated in the leaves is more complex than in the root, as maybe indicated by the pattern of transcription factor related GO terms in the leaf, or that there are a more dynamic changes (or even more ‘noise’) in leaf as opposed to root gene expression, as maybe indicated by the lower association of leaf-u-DEGs compared to d-DEGs with GO terms, is not clear (Fig 4). However, it was a general observation that individual leaf u-DEGs were less likely to also be present in more than one genotype than other classes of DEGs, or, to put it differently, the sets of leaf u-DEGs were more genotype-specific than leaf d-DEGs and root u- and d-DEGs.

Another general observation was that the number of DEGs enriching the common set of GO terms shared by all genotypes was between c. 5–15% of the total (Fig 4). If we interpret GO terms as reflecting biological programmes this implies that the same biological programmes are enacted largely by different sets of genes in different genotypes. While this is less true for photosynthesis than other groups and, more generally, less true for GO terms enriched by root u-DEGs compared to leaf u-DEGs, it does tend to imply alternative strategies at the gene expression level, involving different degrees of complementarity and redundancy, maybe established as a consequence of the diverse origins of the genotypes under study [59–61]. However, it is also worth observing that while this diversity may be a contributing factor, there was no obviously greater similarity between the two *L*. *perenne* genotypes than any of the other pairwise comparisons. Following on from this, in trying to manipulate quantitative traits, such as drought tolerance, this relative lack of uniformity in the sets of genes which develop the relevant biological programmes does present a challenge in terms of predicting trait outcomes from plant crosses–and particularly so for population improvement in outbreeding, genetically heterogeneous species such as the ryegrasses and fescues. An increasingly widely adopted approach to addressing this challenge in forage grasses is to use genomic selection, which has been implemented, at least on an experimental basis, in a number of reported studies [62–67]. It would be interesting to see if, given the differences between leaf and root in the consistency of the direction of regulation of DEGs, if markers derived from leaf- or root-specific genes carry equal predictive abilities. Currently, we are generating pairwise crosses between individual genotypes used in the present study and we hope to be able to test aspects of this in the derived experimental families.

On a similar theme, this greater conservation of direction of gene expression across genotypes also suggests interesting targets for biotechnological experimentation. S7 Table describes 5 DEGs that were contributing to the enrichment of the transcription factor GO terms in the root that were present across all 4 genotypes. Of these, 3 were directly annotated as C-class heat stress transcription factors and 1 as a predicted protein which BLAST searches indicated was also likely to be a C-class heat stress transcription factor. C-class heat stress transcription factors are monocot specific-types the roles of which are much less well understood than A- and B-classes [68]. However, studies in *F*. *arundinacea* [69] and rice [70] have indicated a functional role for C-class heat-stress transcription factors in the abiotic stress response. The fifth conserved root u-DEG in the transcription factor class was annotated as a stem-specific protein TSJT1. As with the C-class heat-stress transcription factors, the precise role of this gene is somewhat of an enigma, though it has been associated with the drought response in rice [71] as well as the negative regulation of internode development in castor bean [72]. However, the conservation of the differential regulation in the roots of these 5 transcription factors in all 4 genotypes suggests that further investigation using gene manipulation approaches is merited.

## Conclusions

While a number of studies have looked at gene expression differences in response to drought from the angle of candidate gene identification, we have taken a novel approach in focussing on similarities, rather than differences, in patterns of differential gene expression in leaves and roots of 4 diverse *Lolium/Festuca* perennial forage grass genotypes. Our study has indicated that: 1) while many biological programmes (GO terms) are conserved across the 4 genotypes, the degree to which the DEGs which are enriching the same GO terms are conserved is limited. Thus, the 4 genotypes may be enacting the same biological programmes through, in part, different sets of genes; 2) across the 4 genotypes, DEGs are more highly conserved in roots as compared to leaves, particularly in comparison to u-DEGs in the leaves. Together, these findings illustrate some of the gene expression challenges associated with trait combining in grass breeding, but also suggest possibilities for application both in applied genetics and biotechnological evaluation.

## Supporting information

S1 Table *Lolium perenne* OmicsBox annotation file. (XLSX)

S2 Table Sequencing and mapping rate values for the different genotypes. (DOCX)

S3 Table Transcriptome statistics and completeness. (DOCX)

S4 Table Complete lists of differentially expressed genes (DEGs) in leaves and roots for each genotype across the AR and TC comparison points. (XLSX)

S5 Table GO terms present for all 4 genotypes across the trend and expression category sets and/or the core set in the leaf. (XLSX)

S6 Table GO terms present for all 4 genotypes across the trend and expression category sets and/or the core set in the root. (XLSX)

S7 Table Common heat-stress transcription factors in leaves and roots. (XLSX)

S1 Fig Principal component analysis of the 4 leaf and root transcriptome replicates extracted from the 4 genotypes at the EWC sampling points. (PPTX)

S2 Fig Proportions of DEGs enriching the same GO groups which are common to all 4 genotypes. (PPTX)

S1 Results Expanded information on: A) pre-processing, mapping, and quality of sequencing and replicates; B) Significance criteria for the identification of DEGs; c) KEGG enzyme activities.
(DOCX)

S1 File (DS_STORE)
