# Supplementary material for: A comparison of shared patterns of differential gene expression and gene ontologies in response to water-stress in roots and leaves of four diverse genotypes of Lolium and Festuca spp. temperate pasture grasses
Source: PLoS One. 2021 Apr 8;16(4):e0249636. doi: 10.1371/journal.pone.0249636 (PMC8031407; doi:10.1371/journal.pone.0249636)
Supplement: S2 Table — (DOCX) [file pone.0249636.s002.docx]

**S2 Table.** Numbers and types of RNAseq reads and their mapping rates for the different genotypes. Reads indicate the combined number from all replicates at each estimated water content (EWC) point. Mapping rates indicate the minimum and maximum values (min/max). Mapped reads are available at the European Nucleotide Archive (ENA) under the study number PRJEB40944 (<https://www.ebi.ac.uk/ena/browser/view/PRJEB40944>)

|  |  | **EWC** | | | |
| --- | --- | --- | --- | --- | --- |
|  | **Parameter** | **35%** | **15%** | **5%** | **1%** |
| **P194 leaf** |  |  |  |  |  |
|  | Total reads | 59367203 | 77033395 | 66947826 | 77925170 |
|  | Clear reads | 58730180 | 76160926 | 66265870 | 77094084 |
|  | Paired Reads | 55284427 | 71486438 | 62212922 | 72426890 |
|  | Mapping rate (%) | 67/65 | 67/66 | 65/64 | 62/63 |
| **P194 root** |  |  |  |  |  |
|  | Total reads | 116079678 | 145613091 | 108082113 | 74142464 |
|  | Clear reads | 114719569 | 144044533 | 104784856 | 73290398 |
|  | Paired Reads | 107145976 | 135224484 | 98505515 | 66514621 |
|  | Mapping rate (%) | 64/54 | 64/63 | 64/59 | 60/52 |
| **Bf11 leaf** |  |  |  |  |  |
|  | Total reads | 50680002 | 59944647 | 57841049 | 55384761 |
|  | Clear reads | 50353992 | 59549552 | 57481115 | 55034505 |
|  | Paired Reads | 48191039 | 57048360 | 55212135 | 52848221 |
|  | Mapping rate (%) | 62/59 | 60/58 | 60/57 | 56/54 |
| **Bf11 root** |  |  |  |  |  |
|  | Total reads | 72230401 | 82950331 | 62016225 | 61752576 |
|  | Clear reads | 71892769 | 82573133 | 61071743 | 61459078 |
|  | Paired Reads | 69582627 | 80050792 | 60136752 | 59435891 |
|  | Mapping rate (%) | 56/52 | 56/54 | 55/37 | 51/49 |
| **Ba99 leaf** |  |  |  |  |  |
|  | Total reads | 65689081 | 71711294 | 65706676 | 54883056 |
|  | Clear reads | 65197807 | 71215612 | 65399511 | 60694245 |
|  | Paired Reads | 61956576 | 68033044 | 63050125 | 58793784 |
|  | Mapping rate (%) | 77/76 | 77/75 | 76/55 | 74/73 |
| **Ba99 root** |  |  |  |  |  |
|  | Total reads | 97958728 | 83435552 | 77065035 | 69484955 |
|  | Clear reads | 97307631 | 82873944 | 75952124 | 69038373 |
|  | Paired Reads | 93129446 | 79412235 | 73467032 | 65740404 |
|  | Mapping rate (%) | 76/75 | 76/75 | 75/74 | 74/72 |
| **Ba12 leaf** |  |  |  |  |  |
|  | Total reads | 100480254 | 106769471 | 84185001 | 93441188 |
|  | Clear reads | 99089807 | 105329582 | 82922973 | 92196703 |
|  | Paired Reads | 92106159 | 98092297 | 76476576 | 85736991 |
|  | Mapping rate (%) | 77/76 | 76/75 | 76/75 | 73/71 |
| **Ba12 root** |  |  |  |  |  |
|  | Total reads | 106866242 | 130544293 | 100926612 | 91012941 |
|  | Clear reads | 106163389 | 129736078 | 97874650 | 89673233 |
|  | Paired Reads | 101691944 | 124639748 | 93775139 | 82949016 |
|  | Mapping rate (%) | 76/75 | 76/75 | 76/75 | 73/69 |
