# Supplementary material for: A comparison of shared patterns of differential gene expression and gene ontologies in response to water-stress in roots and leaves of four diverse genotypes of Lolium and Festuca spp. temperate pasture grasses
Source: PLoS One. 2021 Apr 8;16(4):e0249636. doi: 10.1371/journal.pone.0249636 (PMC8031407; doi:10.1371/journal.pone.0249636)
Supplement: S3 Table — (DOCX) [file pone.0249636.s003.docx]

**S3 Table. Numbers of transcripts and their completeness.** Numbers of transcripts on the consensus transcriptome (derived using the -*-merge* option in Stringtie [33] for each of the genotypes, for both leaf and root tissues. The completeness of the different transcriptomes as per BUSCO [34] (see Materials and Methods) is also shown where the percentage of complete, fragmented and missing core genes is indicated.

| **LEAF** |  | **BUSCO** | | |
| --- | --- | --- | --- | --- |
|  | **# Transcripts** | **Complete** | **Partial** | **Missing** |
| p194 | 56105 | 77.01 | 9.44 | 13.54 |
| Bf11 | 54406 | 75.63 | 9.51 | 14.86 |
| Ba99 | 57887 | 82.15 | 7.15 | 10.69 |
| Ba12 | 64836 | 83.13 | 7.71 | 9.17 |
| **ROOT** | **# Transcripts** | **Complete** | **Partial** | **Missing** |
| p194 | 69682 | 81.53 | 9.24 | 9.23 |
| Bf11 | 58432 | 76.18 | 11.39 | 12.43 |
| Ba99 | 66877 | 83.19 | 8.13 | 8.68 |
| Ba12 | 73556 | 86.94 | 7.15 | 5.90 |
