# Supplementary material for: A comparison of shared patterns of differential gene expression and gene ontologies in response to water-stress in roots and leaves of four diverse genotypes of Lolium and Festuca spp. temperate pasture grasses
Source: PLoS One. 2021 Apr 8;16(4):e0249636. doi: 10.1371/journal.pone.0249636 (PMC8031407; doi:10.1371/journal.pone.0249636)

## Slide 1
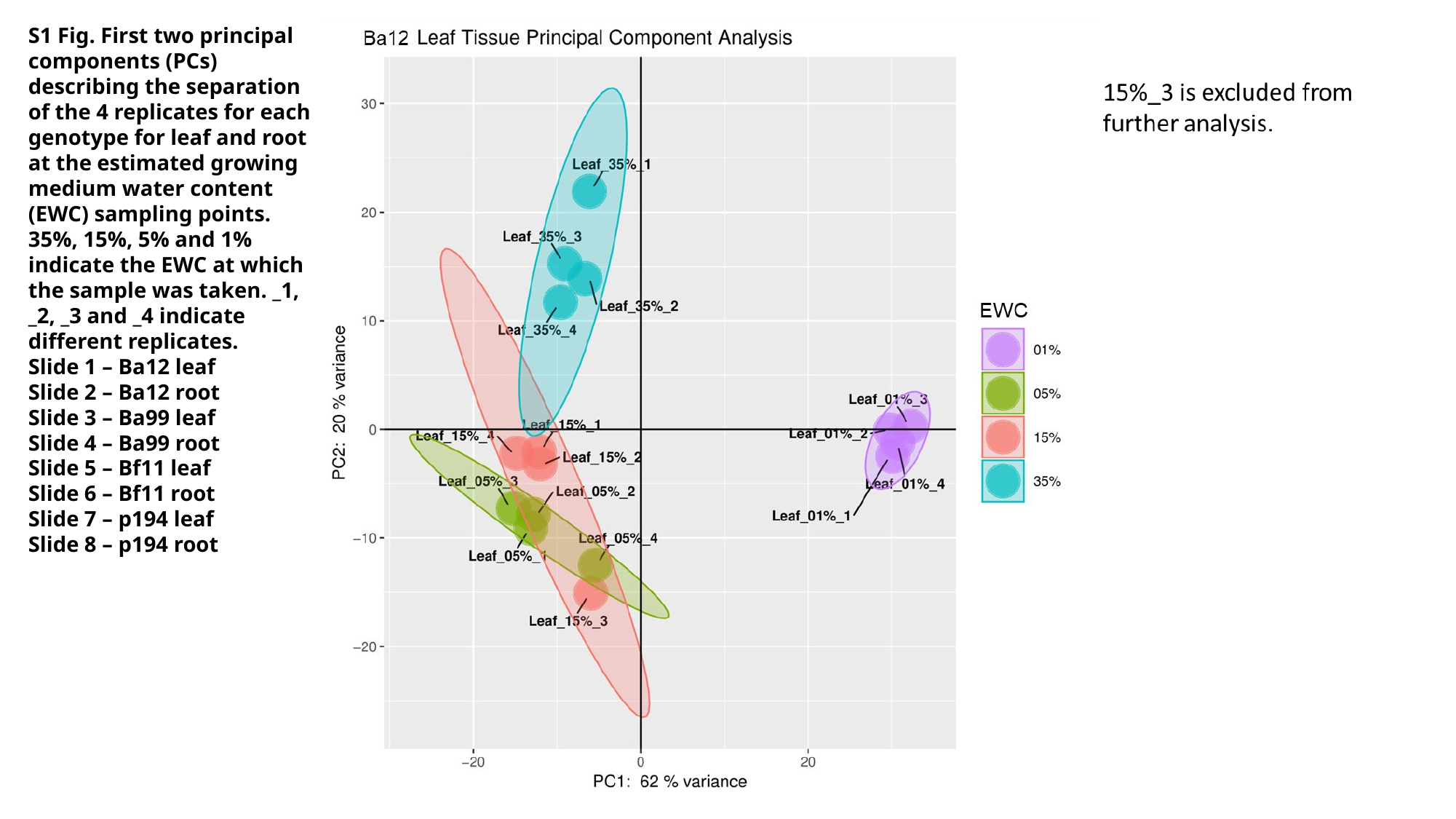

S1 Fig. First two principal components (PCs) describing the separation of the 4 replicates for each genotype for leaf and root at the estimated growing medium water content (EWC) sampling points. 35%, 15%, 5% and 1% indicate the EWC at which the sample was taken. _1, _2, _3 and _4 indicate different replicates.
Slide 1 – Ba12 leaf
Slide 2 – Ba12 root
Slide 3 – Ba99 leaf
Slide 4 – Ba99 root
Slide 5 – Bf11 leaf
Slide 6 – Bf11 root
Slide 7 – p194 leaf
Slide 8 – p194 root

## Slide 2
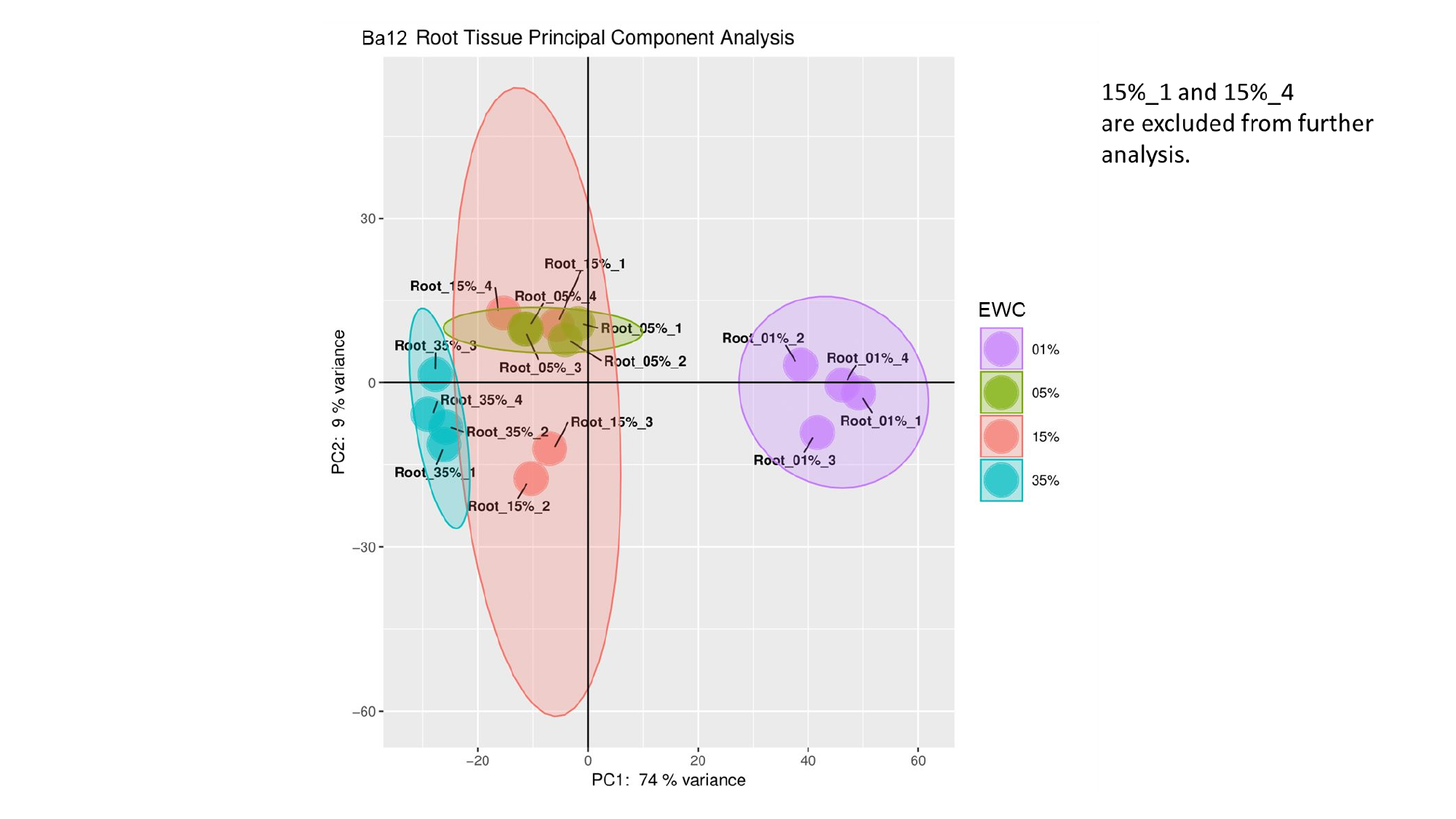

## Slide 3
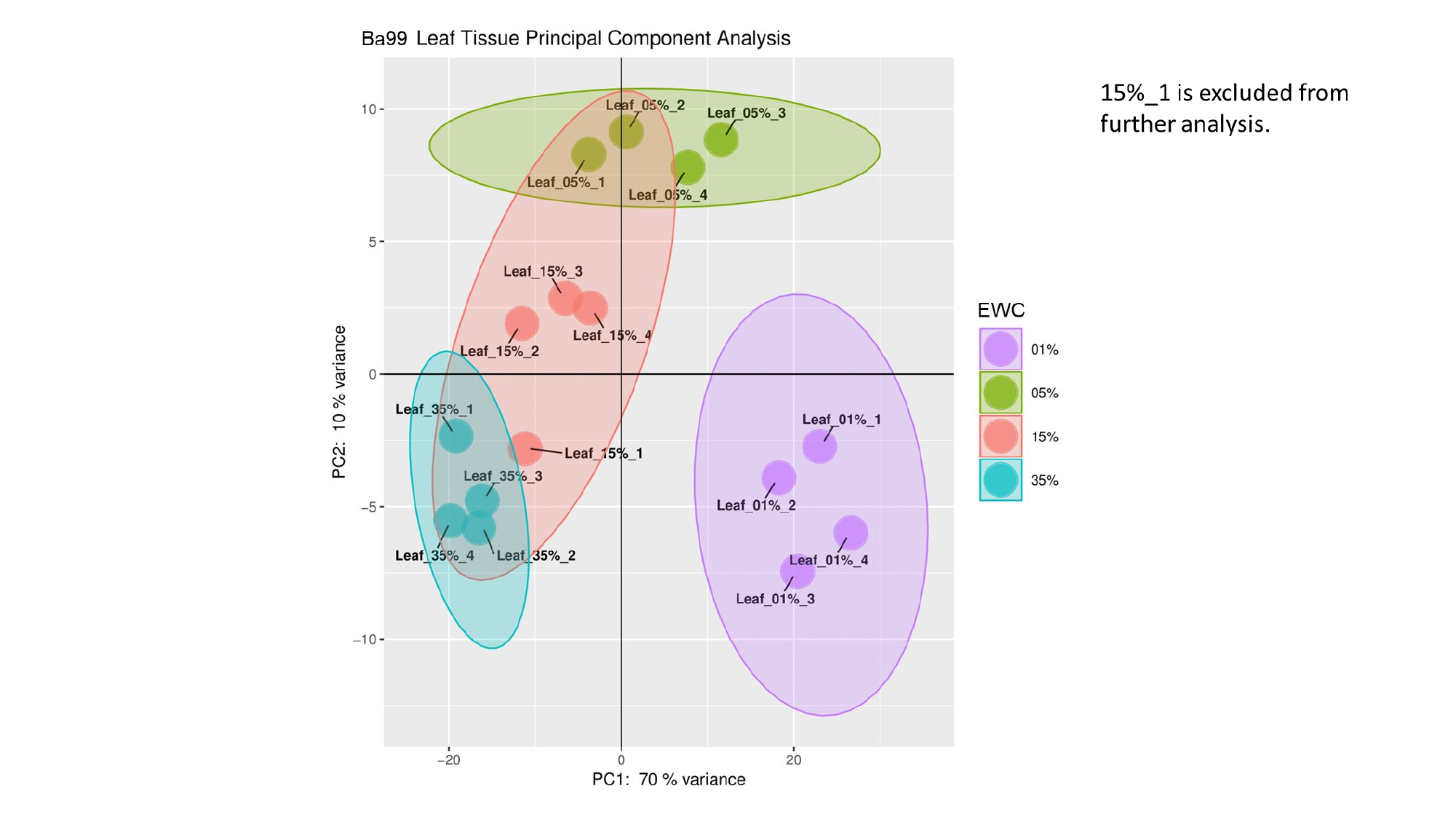

## Slide 4
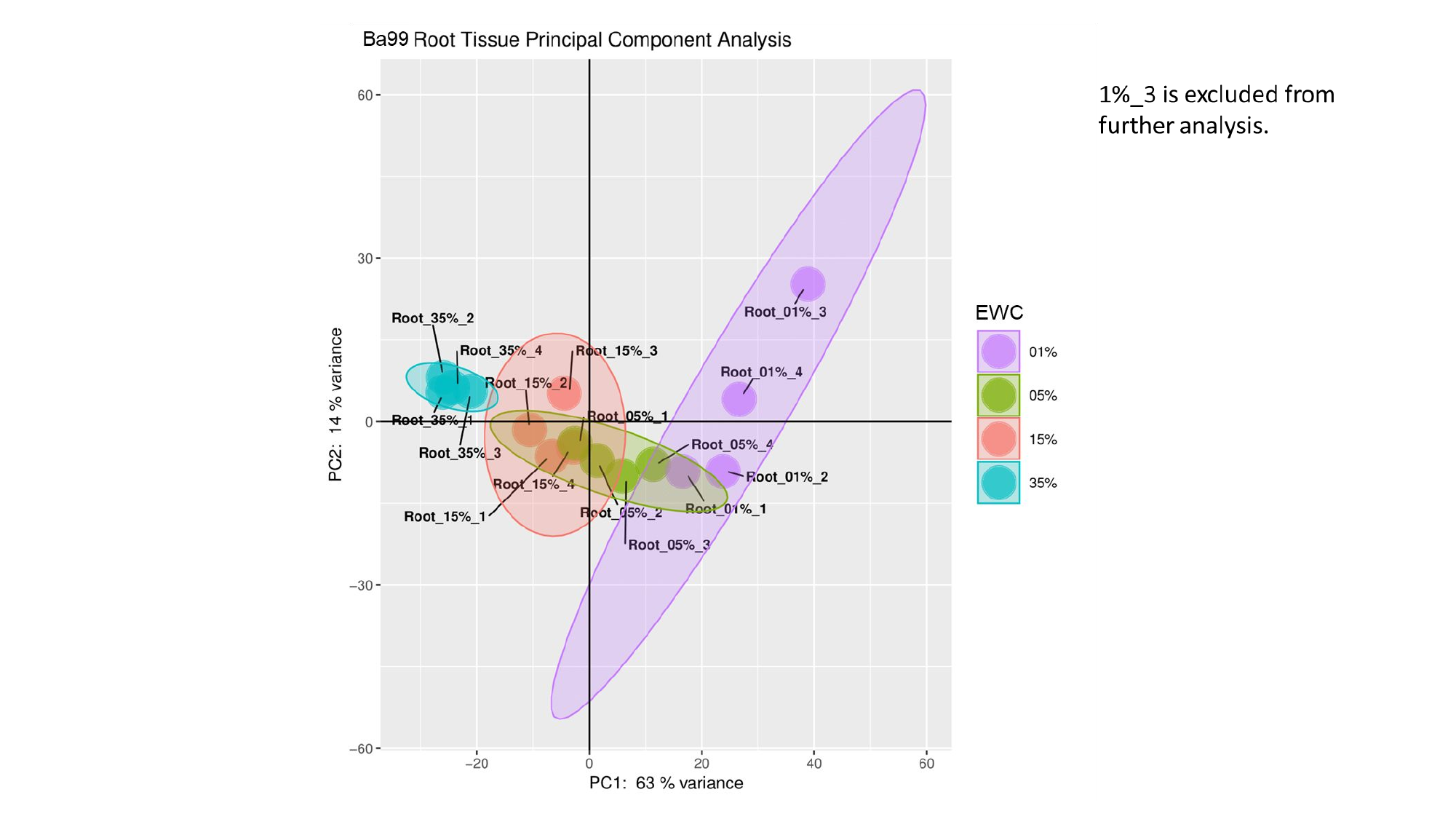

## Slide 5
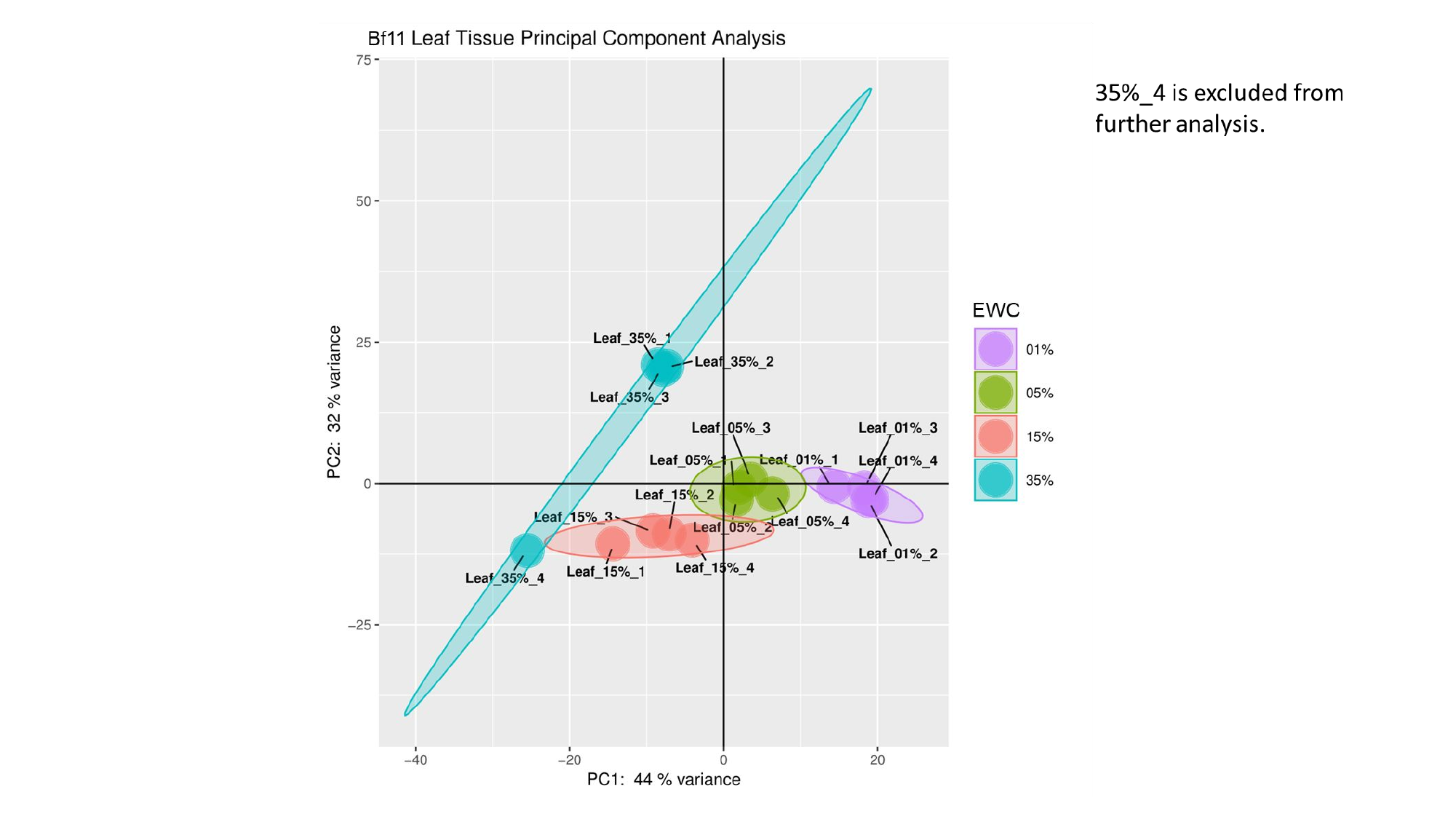

## Slide 6
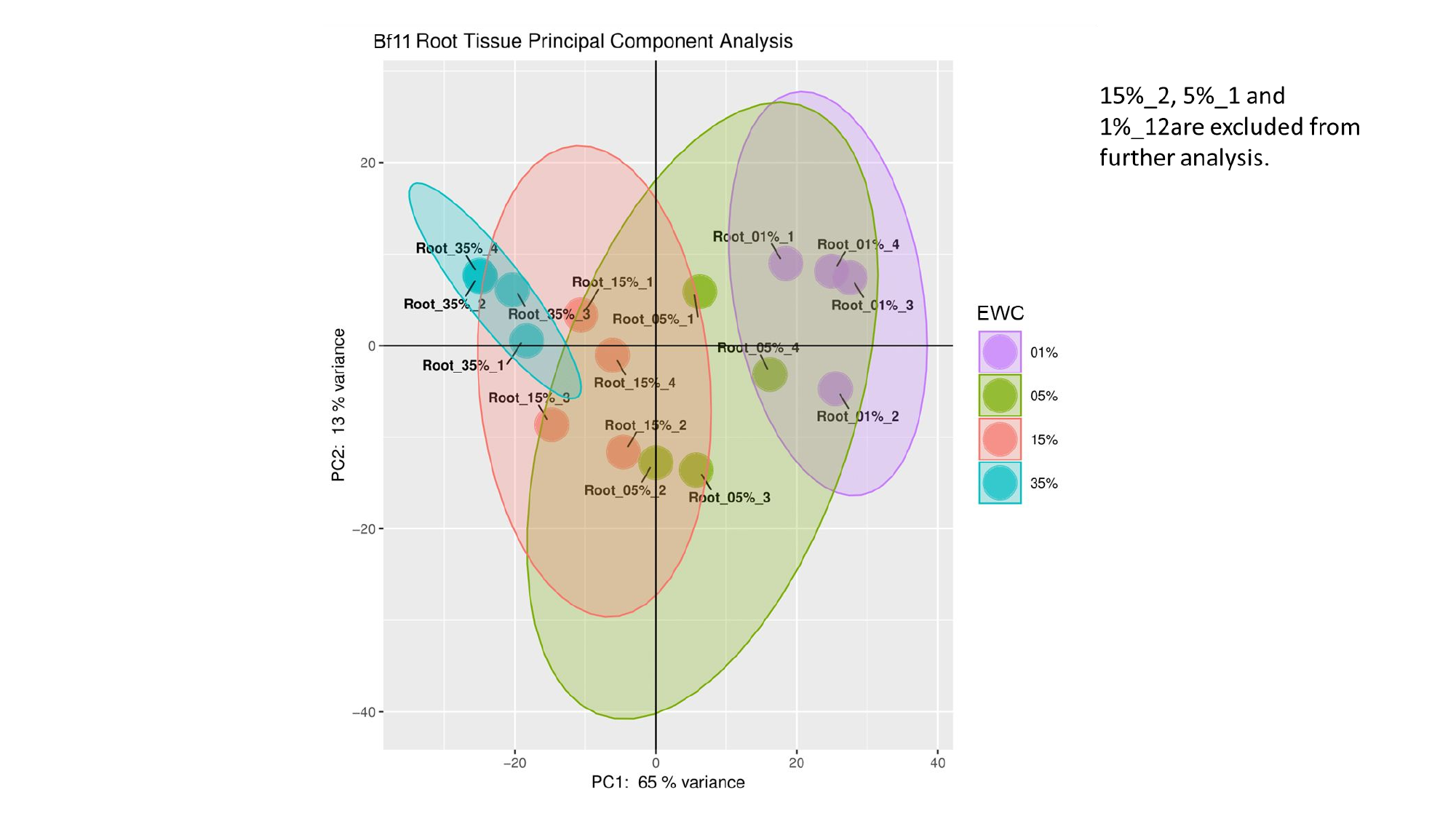

## Slide 7
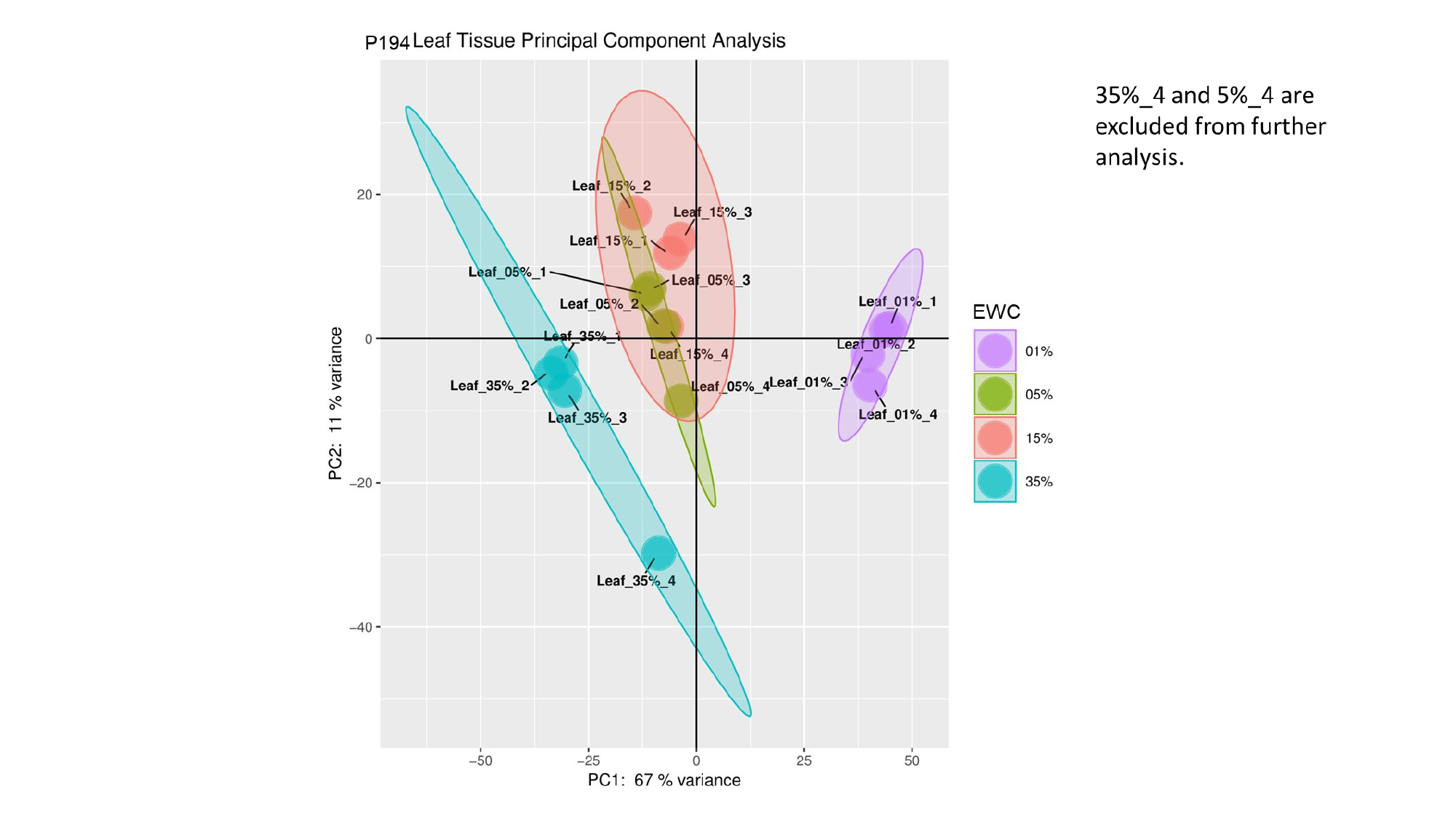

## Slide 8
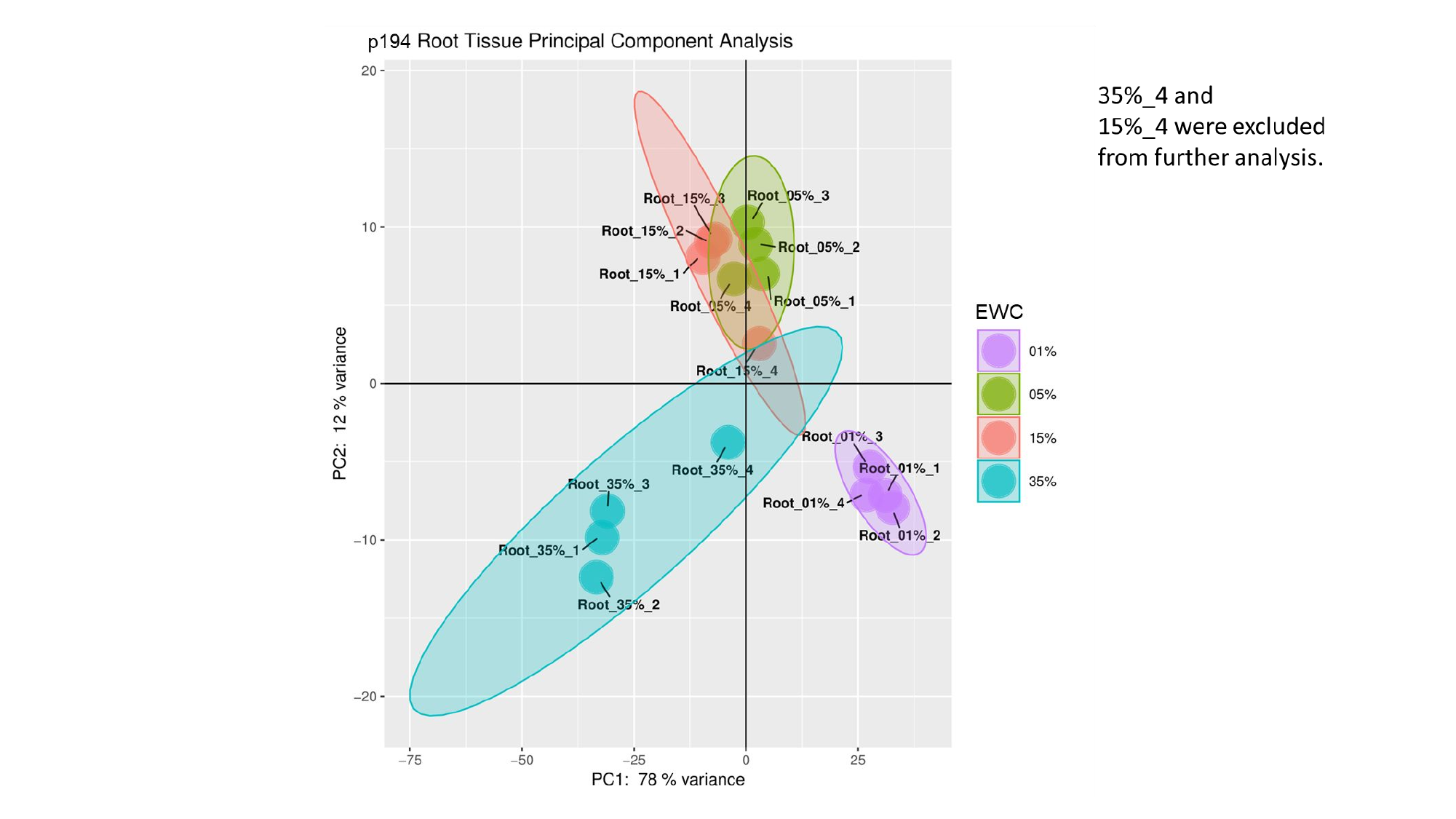

Supplement: S1 Fig — (PPTX) [file pone.0249636.s008.pptx]
