# Supplementary material for: A comparison of shared patterns of differential gene expression and gene ontologies in response to water-stress in roots and leaves of four diverse genotypes of Lolium and Festuca spp. temperate pasture grasses
Source: PLoS One. 2021 Apr 8;16(4):e0249636. doi: 10.1371/journal.pone.0249636 (PMC8031407; doi:10.1371/journal.pone.0249636)
Supplement: S2 Fig — (PPTX) [file pone.0249636.s009.pptx]

## Slide 1
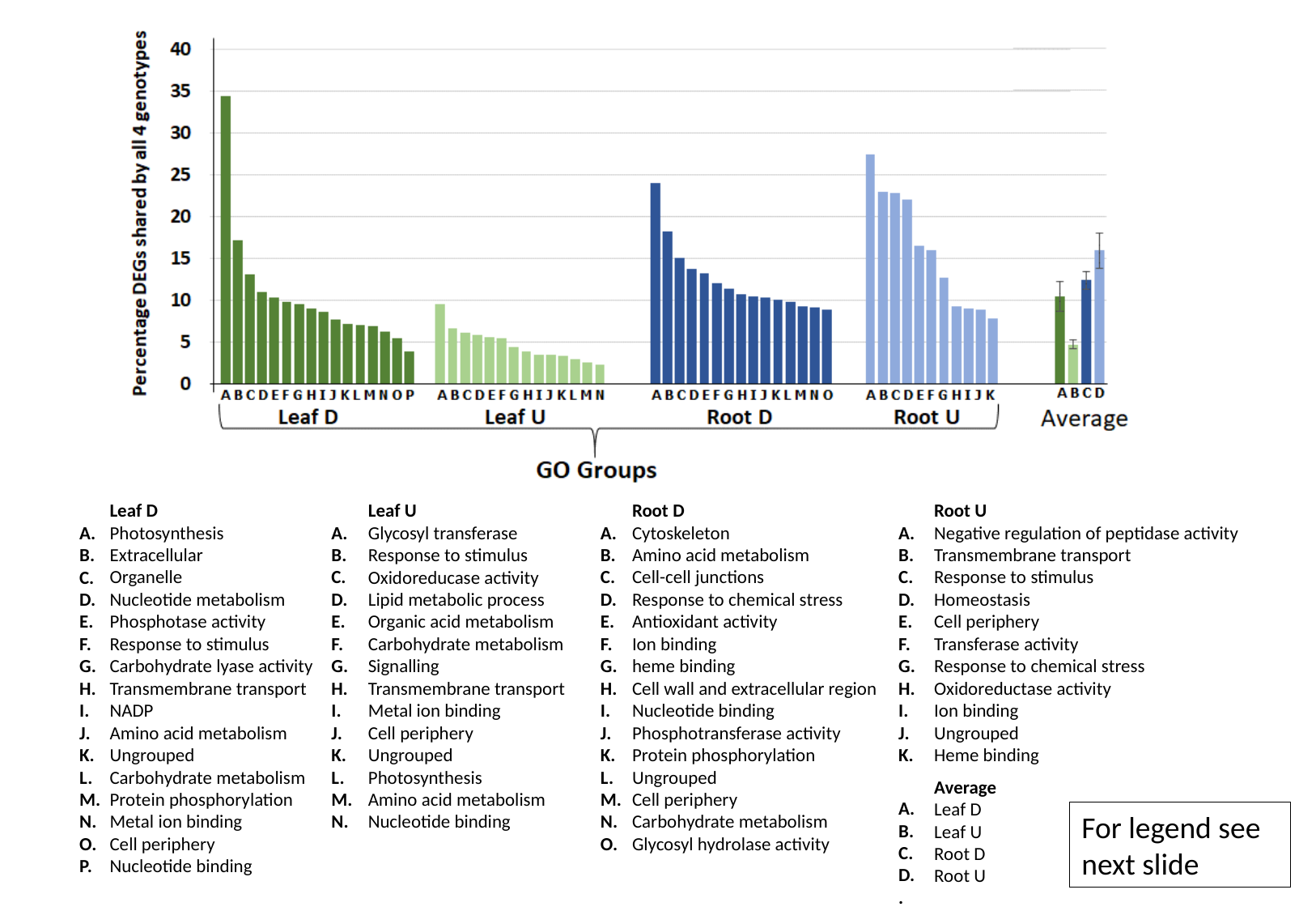

A.
B.
C.
D.
E.
F.
G.
H.
I.
J.
K.
L.
M.
N.
O.
P.
Leaf D
Photosynthesis
Extracellular
Organelle
Nucleotide metabolism
Phosphotase activity
Response to stimulus
Carbohydrate lyase activity
Transmembrane transport
NADP
Amino acid metabolism
Ungrouped
Carbohydrate metabolism
Protein phosphorylation
Metal ion binding
Cell periphery
Nucleotide binding
A.
B.
C.
D.
E.
F.
G.
H.
I.
J.
K.
L.
M.
N.
Leaf U
Glycosyl transferase
Response to stimulus
Oxidoreducase activity
Lipid metabolic process
Organic acid metabolism
Carbohydrate metabolism
Signalling
Transmembrane transport
Metal ion binding
Cell periphery
Ungrouped
Photosynthesis
Amino acid metabolism
Nucleotide binding
A.
B.
C.
D.
E.
F.
G.
H.
I.
J.
K.
L.
M.
N.
O.
Root D
Cytoskeleton
Amino acid metabolism
Cell-cell junctions
Response to chemical stress
Antioxidant activity
Ion binding
heme binding
Cell wall and extracellular region
Nucleotide binding
Phosphotransferase activity
Protein phosphorylation
Ungrouped
Cell periphery
Carbohydrate metabolism
Glycosyl hydrolase activity
A.
B.
C.
D.
E.
F.
G.
H.
I.
J.
K.
Root U
Negative regulation of peptidase activity
Transmembrane transport
Response to stimulus
Homeostasis
Cell periphery
Transferase activity
Response to chemical stress
Oxidoreductase activity
Ion binding
Ungrouped
Heme binding
A.
B.
C.
D.
.
Average
Leaf D
Leaf U
Root D
Root U
For legend see next slide

## Slide 2
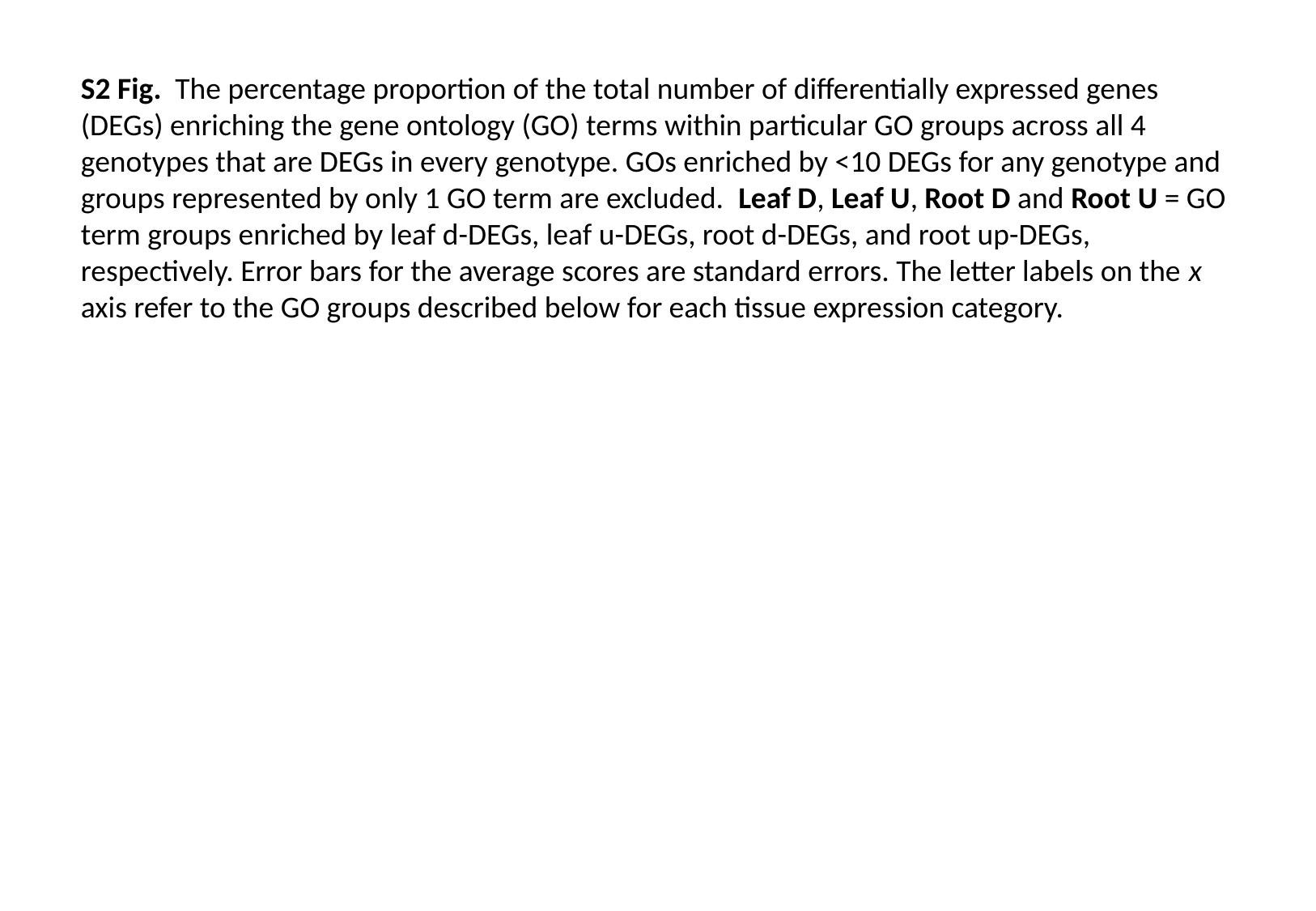

S2 Fig. The percentage proportion of the total number of differentially expressed genes (DEGs) enriching the gene ontology (GO) terms within particular GO groups across all 4 genotypes that are DEGs in every genotype. GOs enriched by <10 DEGs for any genotype and groups represented by only 1 GO term are excluded. Leaf D, Leaf U, Root D and Root U = GO term groups enriched by leaf d-DEGs, leaf u-DEGs, root d-DEGs, and root up-DEGs, respectively. Error bars for the average scores are standard errors. The letter labels on the x axis refer to the GO groups described below for each tissue expression category.
