# Supplementary material for: A comparison of shared patterns of differential gene expression and gene ontologies in response to water-stress in roots and leaves of four diverse genotypes of Lolium and Festuca spp. temperate pasture grasses
Source: PLoS One. 2021 Apr 8;16(4):e0249636. doi: 10.1371/journal.pone.0249636 (PMC8031407; doi:10.1371/journal.pone.0249636)
Supplement: S1 Results — Expanded information on: A) pre-processing, mapping, and quality of sequencing and replicates; B) Significance criteria for the identification of DEGs; c) KEGG enzyme activities. (DOCX) [file pone.0249636.s010.docx]

1. **Pre-processing, mapping, and quality of sequencing and replicates**

Prior to reference-based assembly, the reads were assessed for quality metrics. An average number of reads of 8169084 (p194), 6665617 (Bf11), 7869798 (Ba99), 11012876 (Ba12) in the case of leaf, and 12730956 (p194), 8412689 (Bf11), 9742160 (Ba99), 12595495 (Ba12) in the case of root were trimmed for adapter and low-quality bases and additionally filtered for non-paired reads. The resulting mapping rate for clean reads varied across the genotypes and by tissue, with highest mapping rates for leaf of 67%, 62%, 77% and 77% and for root of 64%, 56%, 76%, and 76% for p194, Bf11, Ba99 and Ba12 respectively (S2 table).

In order to assess the completeness, the transcriptomes at each EWC point were assembled, combined and evaluated using BUSCO. In the case of leaf tissue, completeness’ of transcriptomes (complete and partial genes) were estimated at 86% (p194), 85% (Bf11), 89% (Ba99) and 91% (Ba12). In the case of root tissue, the completeness’ were estimated at 91% (p194), 88% (Bf11), 91% (Ba99) and 94% (Ba12) (S3 Table). PCA results from the raw counts showed the distribution for the 4 replicates for each genotype within each EWC sampling point, and the first two components represented more than 76% of the overall variability (83%,82%: Ba12; 78%, 76%: Bf11; 90%,78%: p194; 77%,80%: Ba99 for root and leaf, respectively (complete details are given in S1 Fig). The PCA indicated strong dissimilarity between 35% and 1% EWC sampling points across all 4 genotypes for both leaf and root. The outliers of each EWC sampling point for both tissues were excluded from further analysis (S1 Fig).

1. **Significance criteria for the identification of DEGs**

Two sets of significance criteria were initially evaluated for DEG calling: i) an absolute value of log_2_ fold change (LFC) >1.2 and an FDR of ≤1%, and ii) LFC >2 and FDR ≤5%. The former criteria were selected for subsequent analyses on the basis of superior functional re-annotation rate. Only transcripts which met the criteria of LFC >1.2 with a FDR of ≤1%, using all the calling methods, i.e. DESeq2, limma-voom and edgeR, were considered to be DEGs for the purposes of this study.

1. **KEGG enzyme activities**

The representation of enzyme codes according to trend and expression category was fairly consistent across the 4 genotypes, with the top 9 most frequently occurring enzyme codes (out of 603 for leaves and 544 for roots) being the same for both leaves and roots and accounting for c. 22% and 33% of the totals for leaves and roots, respectively (Table 1 KEGG). In the leaves, the numbers of enzyme codes represented by d-DEGs and u-DEGs across the 4 genotypes was fairly even, with the exceptions that EC:1.11.1.7 (peroxidase), EC:2.5.1.18 (glutathione transferase) and EC:3.1.3.12 (trehalose-phosphatase) which were associated with d-DEGs and u-DEGs with c. ratios of 3:1, 1:3 and 3:1 respectively. While not detailed in Table 4, EC:2.4.1.15 (alpha,alpha-trehalose-phosphate synthase (UDP-forming)) showed a similar pattern to trehalose-phosphatase, being represented by 16 d-DEGs and 1 u-DEG in the leaf. For the root, the association of d-DEGs and u-DEGs with particular enzyme codes tended to be less even. The 3 most numerously occurring enzyme codes EC:3.6.1.15 (nucleoside-triphosphate phosphatase), EC:1.11.1.7 (peroxidase) and EC:3.6.1.3 (adenosinetriphosphatase) were all represented by d-DEGs:u-DEGs with ratios of c. 3:1. For EC:2.5.1.18 (glutathione transferase), EC:3.1.3.2 (acid phosphatase) and EC:2.7.11.17 (Ca2+/calmodulin-dependent protein kinase) the ratios were c. 2:1 in the opposite direction. As with the leaf, EC:3.1.3.12 (trehalose-phosphatase) and alpha,alpha-trehalose-phosphate synthase (UDP-forming) were predominantly represented by d-DEGS (30:2 and 14:0, d-DEGS:u-DEGS, respectively).

Of the most numerous codes, both EC3.6.1.15 and EC3.6.1.3 (the latter was a subset of the former in terms of the actual d-DEGs) have ATP phosphohydrolase (ATPase) activities and so are universally involved in energy transduction in the cell. Approximately 50% of the d-DEGs associated with EC3.6.1.15 and EC3.6.1.3 (n=117 across all genotypes) had microtubule-associated annotations indicating a reduction in cytoskeletal activity in the root – which was also indicated by the GOs (S7 Table); changes in cytoskeletal organisation in the root has been associated with water stress [1]. EC3.6.1.15 has also been associated with extracellular (apoplastic) ATPase (eATPase) activity and so mediation of eATP signalling. The release of eATPs, and so the potential role of eATPase, has been particularly associated with biotic stress and wounding, but abiotic stresses such as osmotic shock and mechanical pressure have also been implicated [2-4]. The other most frequently occurring enzyme code, EC:1.11.1.7, represents a peroxidase activity that is also associated with the apoplast [5], in this case the target is modification of cell wall lignification and elasticity [6]. In a previous study on perennial ryegrass [7] and a study on the tropical forage grass Brachiaria, using similar experimental designs (Fernandez-Fuentes, unpublished), EC:1.11.1.7 was also found to be disproportionally represented in terms of d-DEGs in the root. The drying of the growing medium will impose new mechanical stresses on the root cell wall and adjustment in cell wall elasticity is likely to be a response. Down-regulation of this enzyme class might be more likely to contribute to increasing the elasticity of cell walls [8] and it will be interesting to see if future work can identify whether this enzyme class plays a generic role in the root’s response to water stress in perennial pasture grasses.

| **Table 1 KEGG**. The ten most frequently occurring enzyme codes amongst the differentially expressed genes in each of leaf and root trend sets. Nine out of the ten codes were common to both leaf and root. | | | | | | | | | | | | | | | | | | | | | | | | | | | |
| --- | --- | --- | --- | --- | --- | --- | --- | --- | --- | --- | --- | --- | --- | --- | --- | --- | --- | --- | --- | --- | --- | --- | --- | --- | --- | --- | --- |
|  |  |  | **Leaf DEGs^1^** | | | | | | | | | | | |  | **Root DEGs^1^** | | | | | | | | | | | |
|  |  |  | **d** | | | | **u** | | | | **i** | | | |  | **d** | | | | **u** | | | | **i** | | | |
| **Tissue** | **Enzyme Code** | **Enzyme Code Description** | **Ba12** | **Ba99** | **Bf11** | **p194** | **Ba12** | **Ba99** | **Bf11** | **p194** | **Ba12** | **Ba99** | **Bf11** | **p194** |  | **Ba12** | **Ba99** | **Bf11** | **p194** | **Ba12** | **Ba99** | **Bf11** | **p194** | **Ba12** | **Ba99** | **Bf11** | **p194** |
| **Leaf/root** | **EC:3.6.1.15** | **nucleoside-triphosphate phosphatase** | 32 | 35 | 13 | 39 | 20 | 20 | 26 | 30 | 2 | - | 6 | 7 |  | 103 | 56 | 57 | 44 | 31 | 7 | 19 | 29 | - | - | - | - |
| **Leaf/root** | **EC:1.11.1.7** | **peroxidase** | 18 | 22 | 15 | 23 | 8 | 5 | 9 | 5 | 3 | - | 2 | 3 |  | 63 | 29 | 28 | 47 | 17 | 14 | 12 | 16 | - | - | - | 1 |
| **Leaf/root** | **EC:3.6.1.3** | **adenosinetriphosphatase** | 17 | 21 | 5 | 18 | 13 | 10 | 17 | 15 | 1 | - | - | 1 |  | 64 | 36 | 37 | 29 | 17 | 6 | 12 | 17 | - | - | - | - |
| **Leaf/root** | **EC:3.1.3.16** | **protein-serine/threonine phosphatase** | 12 | 7 | 6 | 22 | 9 | 9 | 14 | 10 | 2 | - | - | 3 |  | 11 | 7 | 5 | 10 | 19 | 9 | 13 | 14 | - | - | - | - |
| **Leaf/root** | **EC:3.1.1.1** | **carboxylesterase** | 9 | 9 | 6 | 6 | 8 | 9 | 8 | 11 | 3 | - | 1 | 4 |  | 22 | 8 | 6 | 17 | 15 | 13 | 8 | 12 | 1 | - | - | - |
| **Leaf/root** | **EC:3.2.1.26** | **beta-fructofuranosidase** | 7 | 4 | 10 | 3 | 9 | 8 | 9 | 9 | 1 | 1 | 2 | 4 |  | 11 | 6 | 5 | 9 | 4 | 4 | 5 | 4 | - | - | - | - |
| **Leaf/root** | **EC:3.2.1.21** | **beta-glucosidase** | 6 | 4 | 7 | 10 | 8 | 8 | 2 | 7 | 3 | - | 1 | - |  | 11 | 6 | 8 | 9 | 10 | 6 | 5 | 11 | - | - | - | - |
| **Leaf/root** | **EC:2.5.1.18** | **glutathione transferase** | 5 | 1 | 1 | 6 | 15 | 11 | 4 | 10 | 1 | - | - | - |  | 3 | 3 | 3 | 15 | 16 | 6 | 16 | 12 | 1 | - | - | - |
| **Leaf/root** | **EC:3.1.3.2** | **acid phosphatase** | 6 | 5 | 2 | 4 | 9 | 8 | 5 | 7 | - | - | - | - |  | 6 | 3 | 1 | 2 | 7 | 8 | 4 | 5 | - | - | - | - |
| **Leaf** | **EC:2.7.11.17** | **Ca2+/calmodulin-dependent protein kinase** | 7 | 7 | 3 | 10 | 5 | 5 | 3 | 4 | - | - | 1 | - |  | 1 | - | - | 5 | 5 | 1 | 4 | 3 | - | - | - | - |
| **Root** | **EC:3.1.3.12** | **trehalose-phosphatase** | 6 | 8 | 5 | 8 | 2 | - | 3 | 3 | 1 | 1 | 2 | - |  | 6 | 8 | 6 | 10 | **1** | **-** | **1** | **-** | 3 | - | - | - |
| ^1^ d = d-DEGS, u = u-DEGS and i = i-DEGs | | | | | | | | | | | | | | | | | | | | | | | | | | | |

**S1 Results References**

**1.**  Hsiao AS, Wang K, Ho THD. An intrinsically disordered protein interacts with the cytoskeleton for adaptive root growth under stress. Plant Physiol. 2020;183: 570-587.

**2.** Choi J, Tanaka K, Liang Y, Cao Y, Lee Sang Y, Stacey G. Extracellular ATP, a danger signal, is recognized by DORN1 in Arabidopsis. Biochemical Journal. 2014;463: 429-437.

**3.** Jia LY, Bai JY, Guan DD, Sun K, Jiao QS, Feng HQ. Extracellular ATP: a potential molecule regulating the defence response of plants to biotic stresses - a review. Plant Prot Sci. 2016;52: 221-228.

**4.** Wang LM, Stacey G, Leblanc-Fournier N, Legue V, Moulia B, Davies JM. Early extracellular ATP signaling in Arabidopsis root epidermis: a multi-conductance process. Front Plant Sci. 2019;10: 1064.

**5.** Podgorska A, Burian M, Szal B. Extra-cellular but extra-ordinarily important for cells: apoplastic reactive oxygen species metabolism. Front Plant Sci. 2017;8: 1353.

**6.** Meents MJ, Watanabe Y, Samuels AL. The cell biology of secondary cell wall biosynthesis. Annals of Botany. 2018;121: 1107-1125.

**7.** Fradera-Sola A, Thomas A, Gasior D, Harper J, Hegarty M, Armstead I, et al. Differential gene expression and gene ontologies associated with increasing water-stress in leaf and root transcriptomes of perennial ryegrass (*Lolium perenne*). Plos One. 2019;14: e0220518

**8.** Tenhaken R. Cell wall remodeling under abiotic stress. Front Plant Sci. 2015;5: 771.
